# The construction of a novel prognostic prediction model for glioma based on GWAS-identified prognostic-related risk loci

**DOI:** 10.1515/med-2024-0895

**Published:** 2024-03-15

**Authors:** Jie Wei, Yujie Li, Wenqian Zhou, Xiaoya Ma, Jie Hao, Ting Wen, Bin Li, Tianbo Jin, Mingjun Hu

**Affiliations:** College of Life Science, Northwest University, Xi’an 710127, Shaanxi, China; Key Laboratory of Resource Biology and Biotechnology in Western China, Ministry of Education, Northwest University, Xi’an 710069, Shaanxi, China; Shaanxi Provincial Key Laboratory of Biotechnology, Northwest University, Xi’an 710069, Shaanxi, China; Biomedicine Key Laboratory of Shaanxi Province, Northwest University, Xi’an 710069, Shaanxi, China; School of Medicine, Northwest University, Xi’an 710127, Shaanxi, China; Department of Neurosurgery, Xi’an Chest Hospital, Xi’an 710100, Shaanxi, China; Department of Neurosurgery, Xi’an Chang’an District Hospital, Xi’an 710118, Shaanxi, China

**Keywords:** glioma prognosis, GWAS, risk loci, prognostic prediction model, LASSO Cox regression, nomogram

## Abstract

**Backgrounds:**

Glioma is a highly malignant brain tumor with a grim prognosis. Genetic factors play a role in glioma development. While some susceptibility loci associated with glioma have been identified, the risk loci associated with prognosis have received less attention. This study aims to identify risk loci associated with glioma prognosis and establish a prognostic prediction model for glioma patients in the Chinese Han population.

**Methods:**

A genome-wide association study (GWAS) was conducted to identify risk loci in 484 adult patients with glioma. Cox regression analysis was performed to assess the association between GWAS-risk loci and overall survival as well as progression-free survival in glioma. The prognostic model was constructed using LASSO Cox regression analysis and multivariate Cox regression analysis. The nomogram model was constructed based on the single nucleotide polymorphism (SNP) classifier and clinical indicators, enabling the prediction of survival rates at 1-year, 2-year, and 3-year intervals. Additionally, the receiver operator characteristic (ROC) curve was employed to evaluate the prediction value of the nomogram. Finally, functional enrichment and tumor-infiltrating immune analyses were conducted to examine the biological functions of the associated genes.

**Results:**

Our study found suggestive evidence that a total of 57 SNPs were correlated with glioma prognosis (*p* < 5 × 10^−5^). Subsequently, we identified 25 SNPs with the most significant impact on glioma prognosis and developed a prognostic model based on these SNPs. The 25 SNP-based classifier and clinical factors (including age, gender, surgery, and chemotherapy) were identified as independent prognostic risk factors. Subsequently, we constructed a prognostic nomogram based on independent prognostic factors to predict individualized survival. ROC analyses further showed that the prediction accuracy of the nomogram (AUC = 0.956) comprising the 25 SNP-based classifier and clinical factors was significantly superior to that of each individual variable.

**Conclusion:**

We identified a SNP classifier and clinical indicators that can predict the prognosis of glioma patients and established a prognostic prediction model in the Chinese Han population. This study offers valuable insights for clinical practice, enabling improved evaluation of patients’ prognosis and informing treatment options.

## 1 Introduction

Glioma is the most prevalent primary intracranial tumor, comprising approximately 81% of malignant brain tumors [1]. A nationwide population-based study revealed that the global incidence of glioma is 4.67–5.73 cases per 100,000 individuals [2]. Despite its relatively low incidence, glioma’s poor prognosis and survival rate impose a significant economic burden on patients and society [3]. Currently, the standard treatment for glioma involves surgical resection followed by adjuvant chemotherapy and radiotherapy. Patients with glioma still have a very poor prognosis, are prone to relapse, and experience short survival times after treatment, primarily due to the diffuse invasion of the tumor, the vulnerability of the central nervous system, and the impact of various treatments on their function [4]. According to the fifth edition of the WHO Classification of Tumors of Central Nervous System in 2021 (WHO CNS5), gliomas are typically classified into adult-type diffuse gliomas (grades 2, 3, and 4), childhood-type diffuse low-grade gliomas (grade 1), childhood-type diffuse high-grade gliomas (grade 4), localized astrocytomas (grades 2, 3, and 4), glial neurons, and neuronal tumors [5]. In 2016, the WHO included molecular genetic features in the classification of glioma. When there is a discrepancy between the classification results based on histological and molecular genetic features, the genotype is considered more significant than the histological phenotype, emphasizing the importance of molecular genetics in glioma [6]. Some patients diagnosed with low-grade glioma [1] experience slow disease progression, while others may progress to high-grade glioma [4]. Studies have confirmed that the patients with high-grade glioma treated with multimodal strategies have a median survival time of 14–16 months, and the 5-year survival rate of approximately 5% [7,8]. Therefore, it is crucial to discover more reliable biomarkers for glioma prognosis.

Genomic research, such as genome-wide association study (GWAS), plays a vital role in elucidating the pathogenesis of cancers and identifying prognostic markers [9,10]. Previous studies have identified certain genetic variants, such as single nucleotide polymorphisms (SNPs) that are associated with the risk and prognosis of glioma. For instance, GWAS discovered that rs688755 of the *CYP4F12* gene was linked to an elevated risk of glioma [11]. Atkins et al. identified 31 susceptibility genes for glioma according to a transcriptome-wide association study [12]. Guo et al. reported that genetic variants could impact the susceptibility and prognosis of glioma [13]. Zhang et al. showed that *MAML2* genetic variants were associated with the risk and prognosis of glioma [14]. Besides, a case–control study revealed that rs2531995 and rs879620 could significantly increase death risk in patients with high-grade glioma, and rs2230742 and rs2531992 were related to a worse prognosis in glioma [15]. However, majority of these studies concentrate on the correlation between specific loci and the risk or prognosis of glioma, and these findings have not been implemented in clinical prediction models. Prediction model has a critical role in clinical practice to assess prognosis and determine treatment methods. Therefore, it is important to construct prognostic prediction model for glioma based on prognosis-related SNPs.

**Figure 1:**
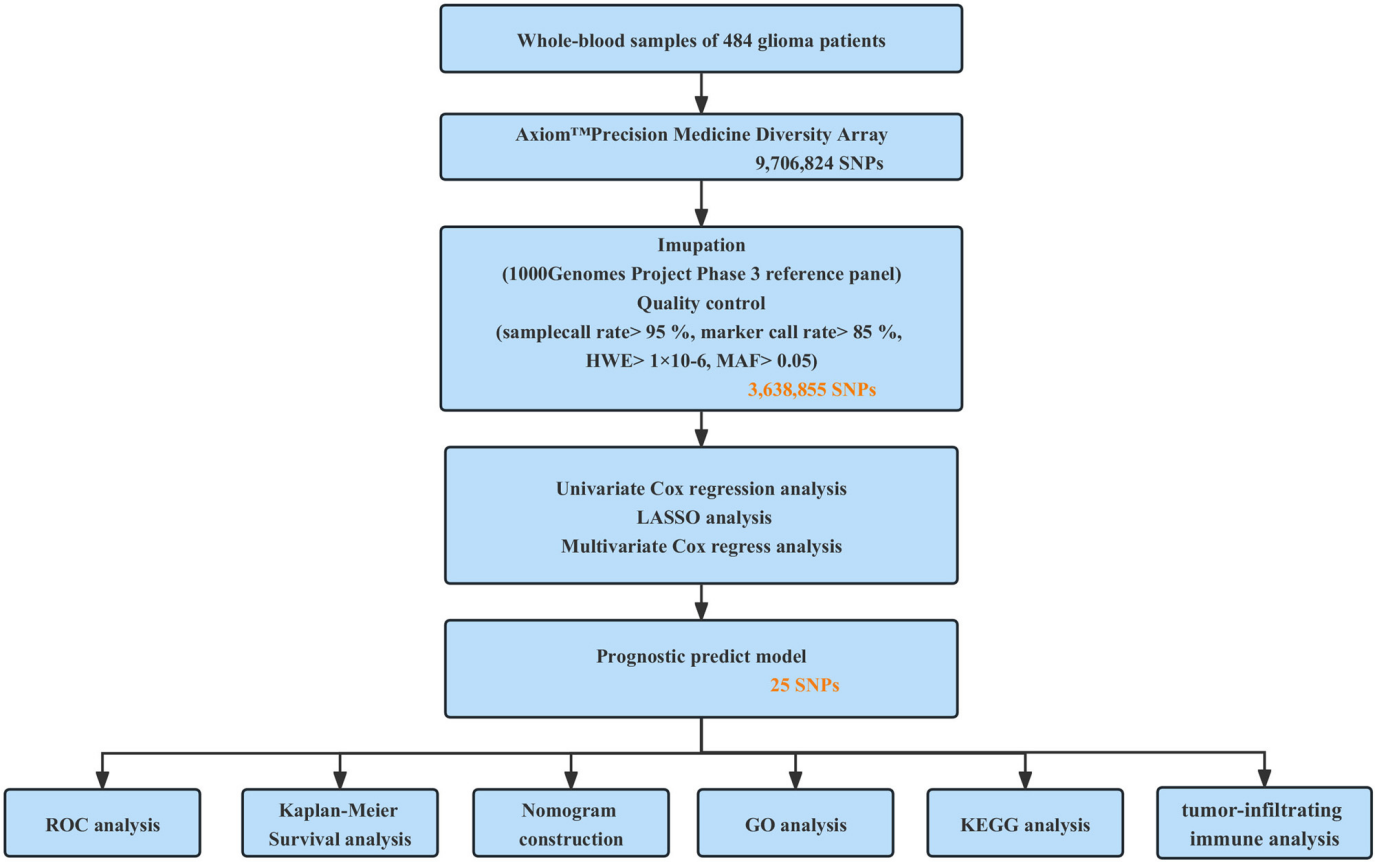
Flowchart of the construction of SNP-related prognostic signature.

In this study, we conducted a GWAS to identify the risk loci associated with glioma prognosis in a cohort of 484 patients. Additionally, we developed a prognostic model for glioma using an SNP-based classifier. Furthermore, to facilitate the clinical application of the prognostic model, we established a prognostic scoring system based on nomogram to predict the 1-year, 2-year, and 3-year survival time of patients. An overview of the workflow is depicted in Figure 1.

## 2 Materials and methods

### 2.1 Study population

This study randomly enrolled 484 adult patients with glioma from Xi’an Chest Hospital. Additionally, all participants have been long-term residents of Shaanxi Province, China, and they belong to an unrelated Han population. Patients were newly diagnosed with glioma through neuropathological examination and confirmed by two neuropathologists based on the criteria established by the World Health Organization (WHO). Moreover, the inclusion criteria for patients were as follows: [1] age >18 years old, [2] no family history of any tumors, including glioma, [3] absence of severe infectious diseases, [4] no history of neurological diseases, [5] no history of malignancies and underlying disease, and [6] no prior chemotherapy or radiotherapy before surgery. In addition, the research objective of our study was clearly communicated, and informed consent was obtained from each participant prior to the commencement of the study. The clinical data of all subjects were collected through medical records, questionnaires, and follow-up procedures, encompassing information such as age, gender, WHO grade, surgery, radiotherapy, and chemotherapy strategies, as well as survival condition (survival or death). The follow-up information for all patients was collected by telephone interviews. Patients were followed up for more than 4 years. The study endpoint, overall survival (OS), was defined as the period from surgery to either death or the last follow-up.


**Ethics approval:** All procedures performed in studies involving human participants were in accordance with the ethical standards of the Xi’an Chest Hospital and Helsinki’s Declaration, and informed consent was obtained from all individual participants included in the study.
**Consent for participate**: Informed written consent was obtained from all participants.

### 2.2 Identification of prognostic-related SNPs with GWAS analysis

Genomic DNA was extracted from whole-blood samples of the patients using a genomic DNA purification kit (GoldMag, Xi’an, China). The quality and concentration of the genomic DNA were assessed using the Nano Drop2000 spectrophotometer, and only the high-quality DNA was utilized for subsequent analysis. Genotyping was performed using the Axiom™Precision Medicine Diversity Array (Thermo Fisher Scientific Inc., Waltham, MA, USA). Genotype calling was conducted using the Affymetrix Gene Titan (Affymetrix, Inc., Santa Clara, CA, USA). Following genotyping, a total of 9,706,824 SNPs were obtained. Prior to imputation, genetic loci exhibiting insertion–deletion (Indel), duplication, copy number variation, and sex chromosome abnormalities were excluded. The quality control (QC) for the remaining loci included the following criteria: sample call rate >95%, minor allele frequency (MAF) >0.05, marker call rate >90%, and Hardy–Weinberg equilibrium (HWE) >5 × 10^−6^. Additionally, principal component analysis (PCA) was conducted to assess population stratification using PLINK 1.9 software [16]. The detailed steps for imputation and QC of these genetic loci were as follows: [1] Imputation was performed using IMPUTE2 software [17] with the 1,000 Genomes Project Phase 3 reference panel [2]. SNPs with a sample call rate <95%, marker call rate <85%, HWE <1 × 10^−6^, MAF <0.05, and allele = 2 were excluded. In the end, a total of 3,638,855 SNPs were obtained. Next, we performed univariate Cox regression analysis to identify SNPs associated with OS and progression-free survival (PFS). We used the “CMplot” package in R (version 4.1.1) to construct quantile–quantile (Q–Q) and Manhattan plots to screen for candidate SNPs with Cox-*p* values less than 5 × 10^−5^ that were associated with OS and PFS [18]. After comparing and overlapping the results, the SNPs associated with glioma prognosis were identified.

### 2.3 Construction of a prognostic prediction model based on SNPs related to glioma prognosis

The SNPs related to prognosis were used to construct the prognostic model. Initially, the least absolute shrinkage and selection operator (LASSO) model, implemented using the “glmnet” package of R software (version 4.1.1) [19], was employed to select the most significant SNPs associated with prognosis. The regression coefficient (*β*) was obtained from the multivariate Cox regression analysis. The risk score (RS) of each patient was calculated by combining the number of risk alleles for the candidate SNPs with their respective regression coefficients. The RS was calculated using the formula: RS = *β*1*G*1 + *β*2*G*2 + *β*3*G*3 + … + *βnGn*, where *βn* represents the regression coefficient of SNP *n* in LASSO Cox regression analysis, *n* is the number of SNP, and *G* is the number of risk alleles for the SNPs. The prognostic model was constructed using the above SNPs, and the cut-off value of the model was determined by analyzing the median RS among all participants. Subsequently, all patients were classified into low-risk (RS < cut-off value) and high-risk groups (RS > cut-off value). Second, a univariate and multivariate Cox regression analyses using “survival” package in R (version 4.1.1) [20] were used for exploratory independent prognostic analysis based on SNP classifier (high risk vs low risk groups) and clinical indicators. Cox regression forest plots were generated using an online database Sangerbox (http://sangerbox.com/AllTools? tool_id = 9730908). Subsequently, Kaplan–Meier survival curves and log-rank tests were performed using the “survminer” package in R (version 4.1.1) [21] to evaluate the survival rates of clinical indicators, as well as the low-risk and high-risk groups, stratified by the SNP classifier.

### 2.4 Establishing a nomogram of the prognostic model based on multiple independent prognostic indicators

A nomogram, incorporating various independent prognostic indicators such as the SNP classifier and clinical indicators, can be utilized to predict patient prognosis. Additionally, a calibration curve was performed to evaluate the clinical applicability of this scoring system by predicting the 1-year, 2-year, and 3-year OS rates. Furthermore, a time-dependent receiver operating characteristic (ROC) curve was constructed to evaluate the diagnostic performance of the nomogram. The area under the curve (AUC) represents the 1-year, 2-year, and 3-year OS probabilities, providing an estimation of the accuracy between the observed and predicted survival probabilities.

### 2.5 Identification of genes related to 25 SNPs in the high- and low-risk groups

RNA sequencing (FPKM) data of 644 glioma patients, including 144 GBM and 500 LGG patients, were downloaded from the TCGA database (https://portal.gdc.cancer.gov/) [22]. Subsequently, the expression data of 25 SNP-related genes (33 genes) were extracted, resulting in the retrieval of 24 genes. The “limma” package in R software (version 4.1.1) [23] is utilized to merge data sets, and the removeBatchEffect () function was applied to correct batch effects by adjusting for gene expression levels. Multivariate Cox regression was performed by combining the combined data set with the survival data, and the RS model was constructed using the gene with a *p* < 0.05 and their respective coefficients. The RS for each patient was calculated using the following formula: RS = *β*1*G*1 + *β*2*G*2 + *β*3*G*3 + … + *βnGn*, where *n* represents the number of prognostic genes, *Gn* is the expression value of gene *n*, and *βn* is the regression coefficient of gene *n* in multivariate Cox regression analysis. Based on the median RS, the samples were divided into a high-risk group and a low-risk group, and a corresponding heat map was generated.

### 2.6 Functional enrichment and tumor-infiltrating immune analysis

The “clusterProfiler” package of R software (version 4.1.1) [24] was used to conduct gene ontology (GO) and Kyoto encyclopedia of genes and genome (KEGG) analyses on the basis of 25 SNPs-related genes. Additionally, the “estimate” package in R (version 4.1.1) was utilized to perform CIBERSORT analysis [25]. The scores of infiltrating immune cells were calculated, and the immune-related pathways in the high- and low-risk groups were evaluated.

### 2.7 Statistical analysis

The clinical data of the study participants were analyzed using SPSS version 25.0. The association between SNPs identified from GWAS results and glioma prognosis was determined using univariate Cox regression models, which calculated hazard ratios (HR) and 95% confidence interval (CI). A Cox-*p* value less than 5 × 10^−5^ indicated a suggestively significant association with prognosis. The potential functions of candidate SNPs were predicted using the RegulomeDB online tool (https://regulome.stanford.edu/regulome-search/) [26]. *p*-Values <0.05 with two-sided tests were considered statistically significant.

## 3 Results

### 3.1 Patients’ characteristics

Table 1 presents the distribution of clinical characteristics among the 484 patients with glioma included in this study, comprising 212 men and 272 women. Among the subjects, 223 (46.07%) glioma patients were aged <40 years, while 261 (53.93%) patients aged ≥40 years. Approximately 62.4% of the patients were classified as being in tumor stage I and II. Surgical treatment was administered to all patients following diagnosis. Out of all patients, 331 patients (68.39%) underwent gross total resection (GTR), while 153 (31.61%) patients underwent near-total resection (NTR) or subtotal resection (STR). Throughout the follow-up period, 432 (89.26%) patients experienced mortality.

**Table 1:** Basic characteristics of the patients with glioma

Variables	*N* (484)
Age (years)	
<40	223 (46.07%)
≥40	261 (53.93%)
Gender	
Male	212 (43.7%)
Female	272 (56.3%)
Grade	
Ⅰ	34 (7.0%)
Ⅱ	268 (55.4%)
Ⅲ	127 (26.2%)
Ⅳ	55 (11.4%)
Histological subtype	
Glioblastoma	31 (6.4%)
Astrocytoma	301 (62.2%)
Oligodendroglioma	13 (2.7%)
Oligodendrocytes astrocytoma	69 (14.3%)
Ependymoma	33 (6.8%)
Others	22 (4.5%)
Surgery	
NTR/STR	153 (31.61%)
GTR	331 (68.39%)
Radiotherapy	
No	51 (10.54%)
Conformal radiotherapy	124 (25.62%)
Gamma knife	309 (63.84%)
Chemotherapy	
No	293 (60.54%)
Yes	191 (39.46%)
Score	
Low risk	242 (50.00%)
High risk	242 (50.00%)
Survival status	
Survival	52 (10.74%)
Death	432 (89.26%)

NTR, near-total resection; STR, subtotal resection; GTR, gross total resection.

### 3.2 Risk loci associated with glioma prognosis identified through GWAS

**Figure 2:**
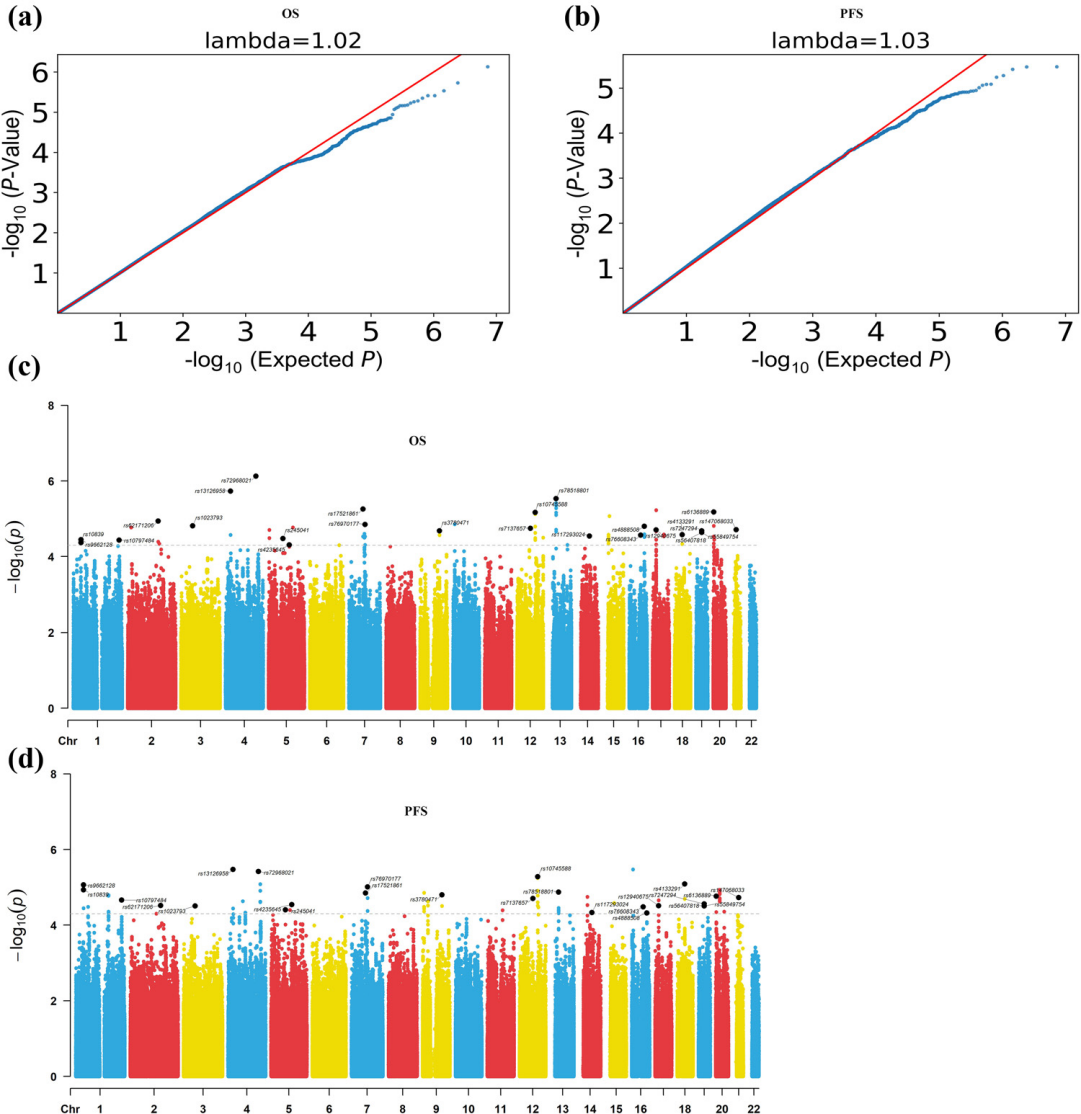
Q–Q plots (a) and (b) and Manhattan plots (c) and (d) of the genome-wide association study. The red line in (c) and (d) indicates the genome-wide suggestive significant threshold of *p* = 5 × 10^−5^.

Figure S1 displays the results of PCA analysis, indicating that our research population is homogeneous and devoid of racial stratification. Following imputation and QC, a Q–Q plot of GWAS *p*-values exhibited that the inflation factor (*λ*) was 1.02 for glioma-OS (Figure 2a) and 1.03 for glioma-PFS (Figure 2b), respectively, indicating that the test statistics followed null expectations and did not exceed the expected small *p*-values. As shown in Figure 2c and d, the Manhattan plot showed the suggestive SNPs on the chromosome location for glioma-OS (Figure 2c) and glioma-PFS (Figure 2d). As shown in Table S1, a total of 106 SNPs were suggestive associated with OS in glioma (*p* < 5 × 10^−5^). Table S2 shows that 115 SNPs were related to PFS in glioma (*p* < 5 × 10^−5^). After analyzing the overlaps, we observed that 57 candidate SNPs with *p* values <5 × 10^−5^ were suggestive identified to be correlated with the prognosis of glioma (Table S3).

### 3.3 Construction of the prognosis prediction model for glioma

**Figure 3:**
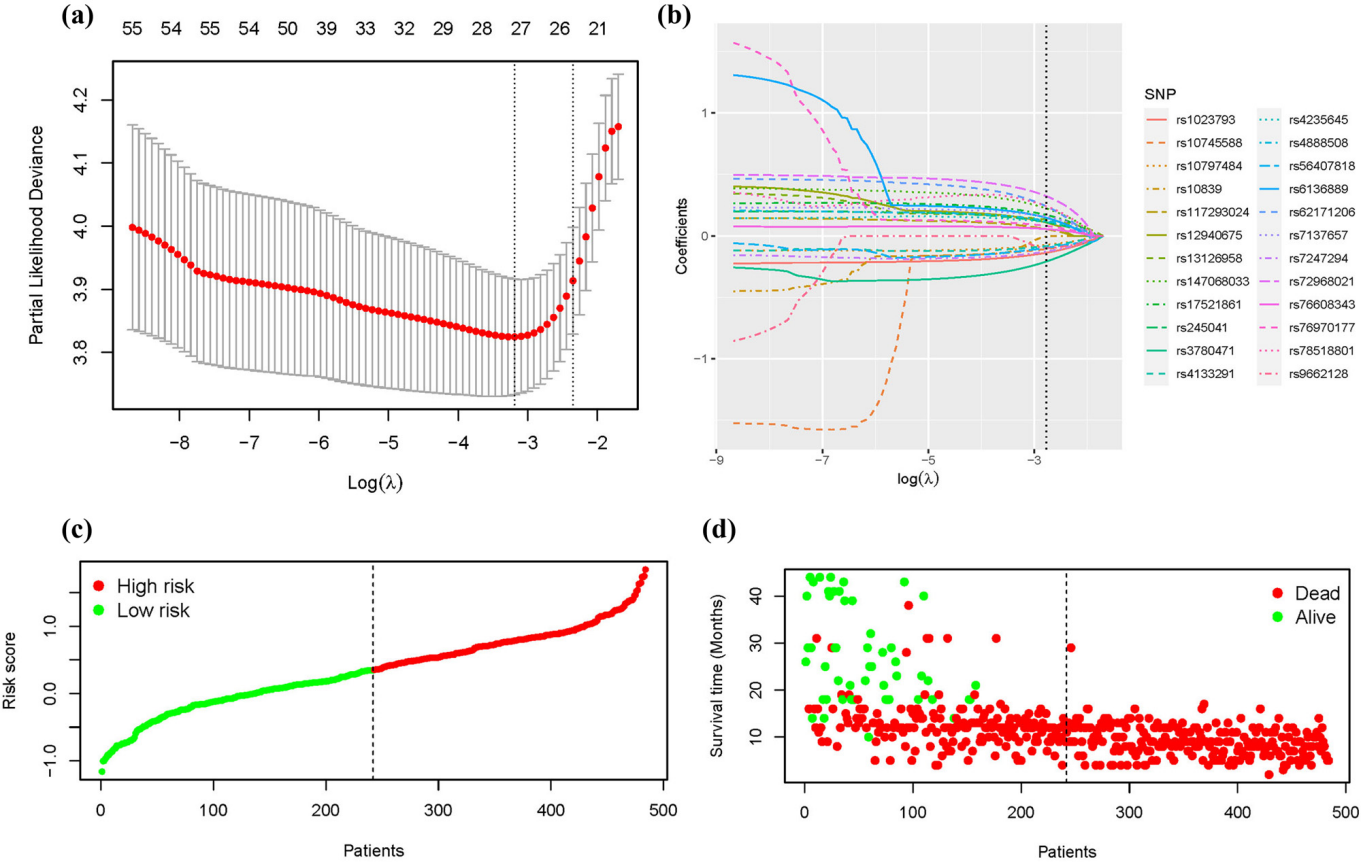
Construction of the glioma prognostic model based on the OS-related SNPs. (a) LASSO coefficient profiles of the 25 OS-related SNPs. (b) Selection of the tuning parameter (*λ*) according to cross-validation curve in the LASSO model. (c) Distribution of RS for glioma. (d) Survival status of patients according to low-risk and high-risk groups.

**Figure 4:**
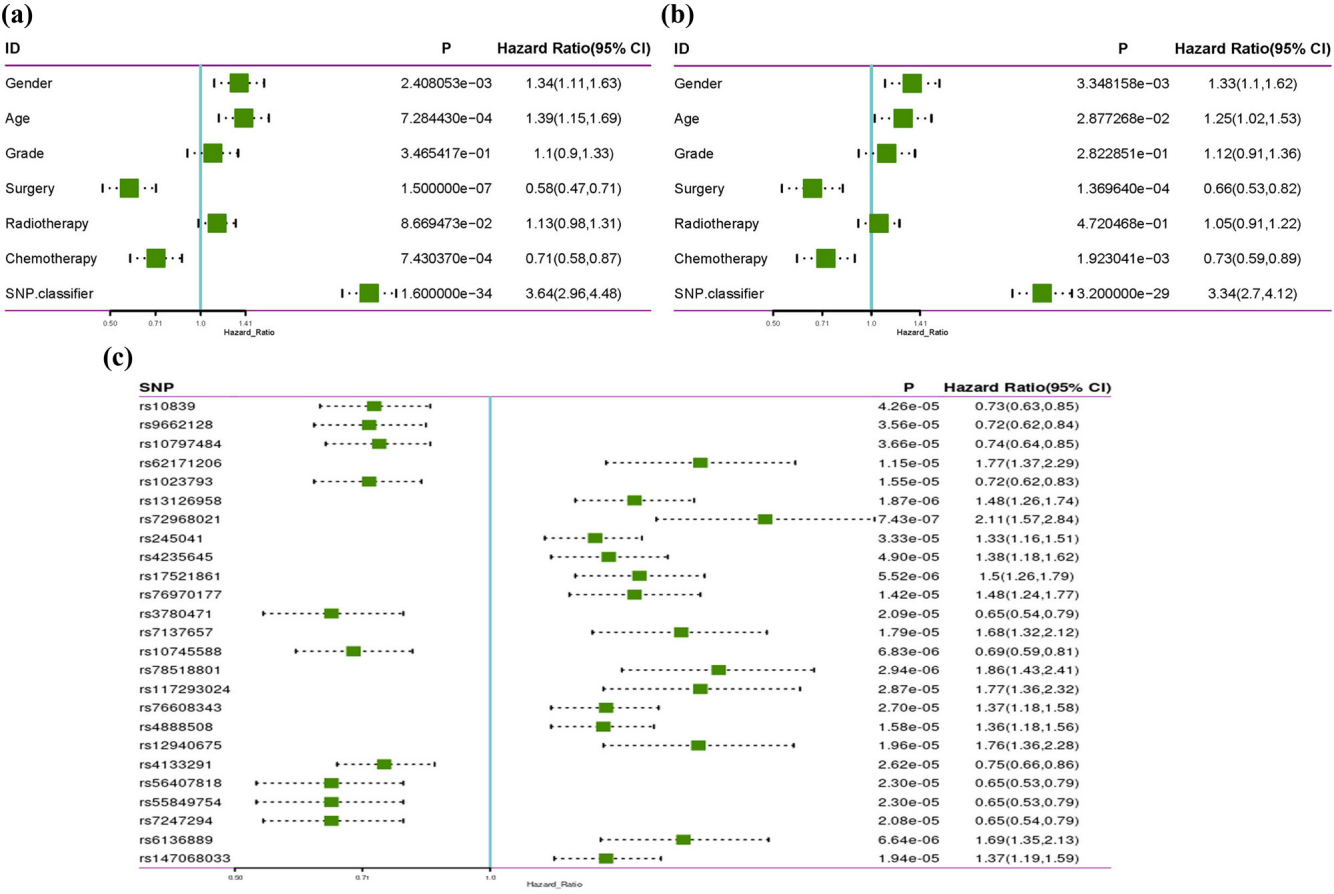
Prognosis value of SNP-based classifier in glioma. (a) Univariate analysis of SNP-based classifier and clinical features in glioma. (b) Multivariate analysis of SNP-based classifier and clinical features in glioma. (c) Association analysis between the 25 SNPs and glioma prognosis.

**Figure 5:**
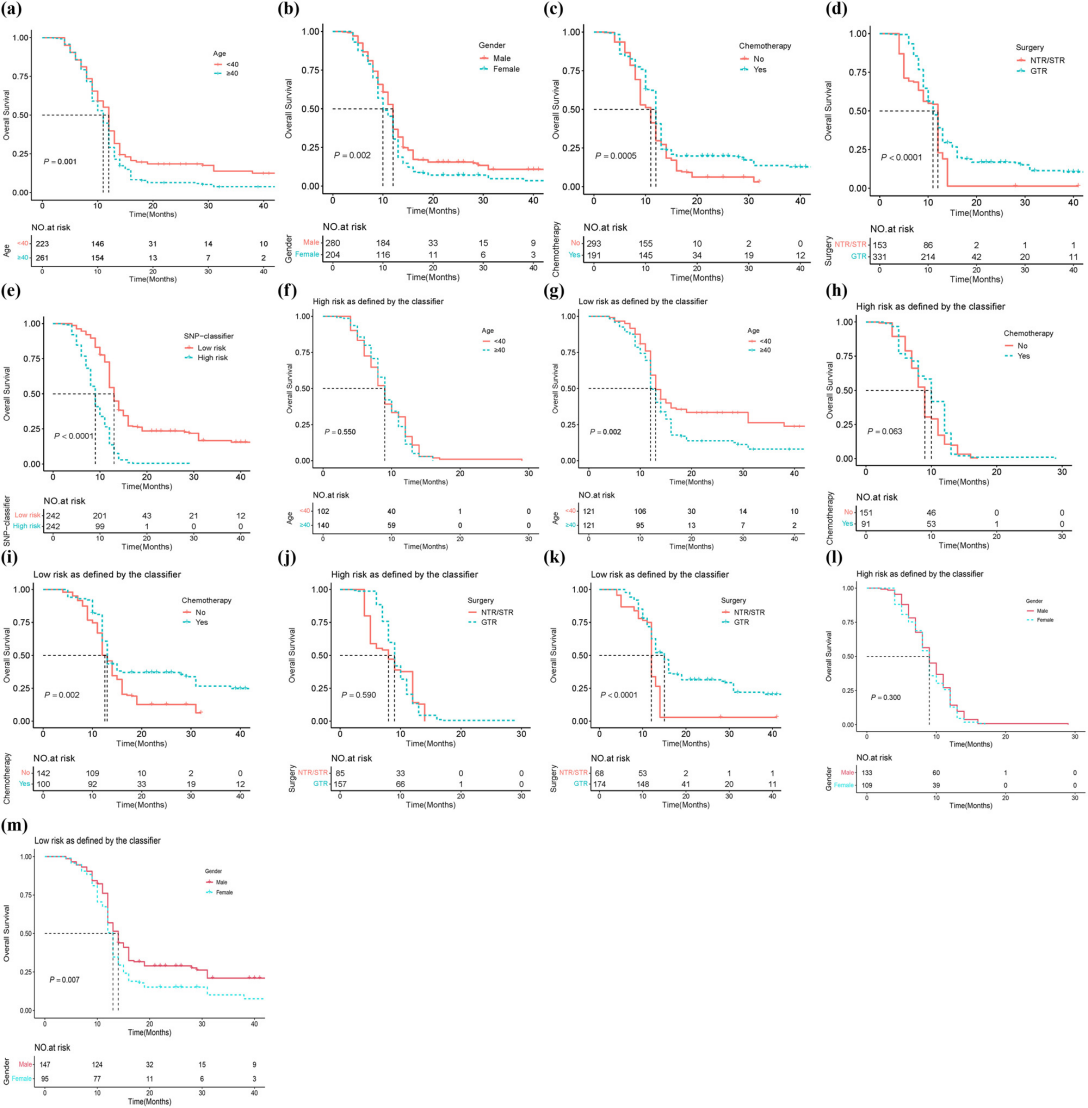
Kaplan–Meier OS analysis of glioma based on prognostic risk factors. (a)–(m) Survival analysis of the signature in patients stratified by age, gender, chemotherapy, surgery, and SNP classifier, high risk as defined by the classifier (age, chemotherapy, surgery, and gender), and low risk as defined by the classifier (age, chemotherapy, surgery, and gender).

GWAS analysis identified 57 SNPs associated with glioma prognosis. We further performed LASSO regression analysis on these 57 SNPs to find out the SNPs with the greatest impact on glioma prognosis. A risk signature was constructed based on the optimal value of *λ* using 25 SNPs as shown in Figure 3a and b. The details of the 25 SNPs are shown in Table 2. The results of RegulomeDB suggested a potential association between these SNPs and the regulation of TF binding, any motif, DNase peak, and Motif hit. The following SNPs were associated with a better prognosis in glioma patients: rs10839 and rs9662128 in *HPCAL4*, rs10797484 in *KCNK1*/*SLC35F3*, rs1023793 in *C3orf67*/*FHIT*, rs3780471 in *TRIM14*, rs10745588 in *LOC101928617*/*CLLU1OS*, rs4133291 in *MIR4318*/*MIR924HG*, and rs56407818, rs55849754, and rs7247294 in *LOC100420587*/*LINC00906* (all HR < 1, *p* < 0.05). The following SNPs were significantly associated with an increased risk of death in glioma: rs62171206 in *GALNT13*, rs13126958 in *CCDC149*, rs72968021 in *DCHS2*, rs245041 in *LINC02056*, rs4235645 in *NUDT12*/*RAB9BP1*, rs17521861 in *GALNT17*, rs76970177 in *SEMA3C*/*LOC105369146*, rs7137657 in *LINC02408*/*DYRK2*, rs78518801 in *CCDC169-SOHLH2*/*SOHLH2*, rs117293024 in *KCNH5*, rs76608343 in *GINS3*, rs4888508 in *CNTNAP4*, rs12940675 in *RAI1*, rs6136889 in *PDYN-AS1*/*STK35*, and rs147068033 in *D21S2088E*/*LINC01689* (all HR >1, *p* < 0.05). Patients with genotypes from the 25 SNPs were divided into two groups based on the median RS: those with RS <0.3507 were classified as low-risk, while those with RS >0.3507 were classified as high risk (Figure 3c). Additionally, Figure 3d illustrates the survival status of glioma patients. The dot plot showed that low-risk patients had a longer survival time, while high-risk patients had a shorter survival time. Univariate and multivariate Cox regression analysis were conducted to investigate the relationship between the SNP-based classifier and clinical characteristics (gender, age, grade, surgery, radiotherapy, and chemotherapy) with the OS of glioma patients. Table 3 and Figure 4a and b demonstrates that gender, age, surgery, chemotherapy, and the SNP classifier were identified as independent risk factors for the prognosis of glioma. Furthermore, Figure 4c reveals a significant association between the 25 SNPs and the prognosis of glioma. Kaplan–Meier survival analysis demonstrated that patients aged <40 years had significantly better OS compared to those aged ≥40 years (*p* = 0.001, Figure 5a). Male patients exhibited significantly better OS compared to female patients (*p* = 0.002, Figure 5b). Patients who received chemotherapy showed improved OS (*p* = 0.0005, Figure 5c). Patients who underwent GTR surgery had significantly better OS compared to those who underwent NTR/STR surgery (*p* < 0.0001, Figure 5d). Additionally, patients in the high-risk group had significantly worse OS compared to the low-risk group (*p* < 0.0001, Figure 5e). Stratification by high- and low-risk groups revealed that patients aged <40 years, males, those who received chemotherapy, and those underwent GTR surgery had better OS in the low-risk group, while no significant differences were observed in the high-risk group (Figure 5f–m).

**Table 2:** Association of genetic loci with prognosis of glioma by LASSO analysis

SNPs	Gene	Chr	BP	Functional consequence	Potential functions	HR (95% CI)	*p*
rs10839	*HPCAL4*	1	39679146	3ʹ-UTR	TF binding or DNase peak	0.73 (0.63–0.85)	4.26 × 10^−5^
rs9662128	*HPCAL4*	1	39684484	Synonymous	TF binding, any motif, DNase peak	0.72 (0.62–0.84)	3.56 × 10^−5^
rs10797484	*KCNK1*; *SLC35F3*	1	233791256	—	TF binding, DNase peak	0.74 (0.64–0.85)	3.66 × 10^−5^
rs62171206	*GALNT13*	2	154257864	Intronic	TF binding or DNase peak	1.77 (1.37–2.29)	1.15 × 10^−5^
rs1023793	*C3orf67*; *FHIT*	3	59341735	Intronic	TF binding or DNase peak	0.72 (0.62–0.83)	1.55 × 10^−5^
rs13126958	*CCDC149*	4	24976807	Intronic	TF binding, DNase peak	1.48 (1.26–1.74)	1.87 × 10^−6^
rs72968021	*DCHS2*	4	154373450	Intronic	Other	2.11 (1.57–2.84)	7.43 × 10^−7^
rs245041	*LINC02056*	5	72640578	Intronic	Motif hit	1.33 (1.16–1.51)	3.33 × 10^−5^
rs4235645	*NUDT12*; *RAB9BP1*	5	104799715	—	Motif hit	1.38 (1.18–1.62)	4.90 × 10^−5^
rs17521861	*GALNT17*	7	71244666	Intronic	TF binding or DNase peak	1.50 (1.26–1.79)	5.52 × 10^−6^
rs76970177	*SEMA3C*; *LOC105369146*	7	81364751	—	TF binding or DNase peak	1.48 (1.24–1.77)	1.42 × 10^−5^
rs3780471	*TRIM14*	9	98085269	Intronic	TF binding, DNase peak	0.65 (0.54–0.79)	2.09 × 10^−5^
rs7137657	*LINC02408*; *DYRK2*	12	67605696	—	Motif hit	1.68 (1.32–2.12)	1.79 × 10^−5^
rs10745588	*LOC101928617*; *CLLU1OS*	12	92250325	—	Other	0.69 (0.59–0.81)	6.83 × 10^−6^
rs78518801	*CCDC169–SOHLH2*; *SOHLH2*	13	36188063	Intronic	TF binding or DNase peak	1.86 (1.43–2.41)	2.94 × 10^−6^
rs117293024	*KCNH5*	14	62952042	Intronic	Motif hit	1.77 (1.36–2.32)	2.87 × 10^−5^
rs76608343	*GINS3*	16	58395747	Intronic	TF binding or DNase peak	1.37 (1.18–1.58)	2.70 × 10^−5^
rs4888508	*CNTNAP4*	16	76438786	Intronic	TF binding, DNase peak	1.36 (1.18–1.56)	1.58 × 10^−5^
rs12940675	*RAI1*	17	17747900	Intronic	TF binding, DNase peak	1.76 (1.36–2.28)	1.96 × 10^−5^
rs4133291	*MIR4318*; *MIR924HG*	18	38898280	—	TF binding or DNase peak	0.75 (0.66–0.86)	2.62 × 10^−5^
rs56407818	*LOC100420587*; *LINC00906*	19	28788472	—	TF binding or DNase peak	0.65 (0.53–0.79)	2.30 × 10^−5^
rs55849754	*LOC100420587*; *LINC00906*	19	28788758	—	TF binding or DNase peak	0.65 (0.53–0.79)	2.30 × 10^−5^
rs7247294	*LOC100420587*; *LINC00906*	19	28789022	—	Other	0.65 (0.54–0.79)	2.08 × 10^−5^
rs6136889	*PDYN-AS1*; *STK35*	20	2064746	—	TF binding, DNase peak	1.69 (1.35–2.13)	6.64 × 10^−6^
rs147068033	*D21S2088E*; *LINC01689*	21	23637454	—	Motif hit	1.37 (1.19–1.59)	1.94 × 10^−5^

SNP: single nucleotide polymorphism, Chr: chromosome, BP: biological position, OS: overall survival, PFS: progression-free survival, HR: hazard ratio, CI: confidence interval.

**Table 3:** Univariate and multivariate analysis of prognostic factors and OS of glioma patients

Variables	Univariate analysis		Multivariate analysis	
	HR (95% CI)	*p*	HR (95% CI)	*p*
Gender (female vs male)	1.34 (1.11–1.63)	0.002	1.33 (1.1–1.62)	0.003
Age (≥40 vs <40)	1.39 (1.15–1.69)	0.001	1.25 (1.02–1.53)	0.029
Grade (III–IV vs I–II)	1.10 (0.90–1.33)	0.347	1.12 (0.91–1.36)	0.282
Surgery (GTR vs NTR/STR)	0.58 (0.47–0.71)	0.000	0.66 (0.53–0.82)	<0.001
Radiotherapy (gamma knife vs no/conformal radiotherapy)	1.13 (0.98–1.31)	0.087	1.05 (0.91–1.22)	0.472
Chemotherapy (yes vs no)	0.71 (0.58–0.87)	0.001	0.73 (0.59–0.89)	0.002
SNP-based classifier (high risk vs low risk)	3.64 (2.96–4.48)	0.000	3.34 (2.7–4.12)	<0.001

### 3.4 Establishment of a nomogram of the prognostic model that included the independent prognostic factors

**Figure 6:**
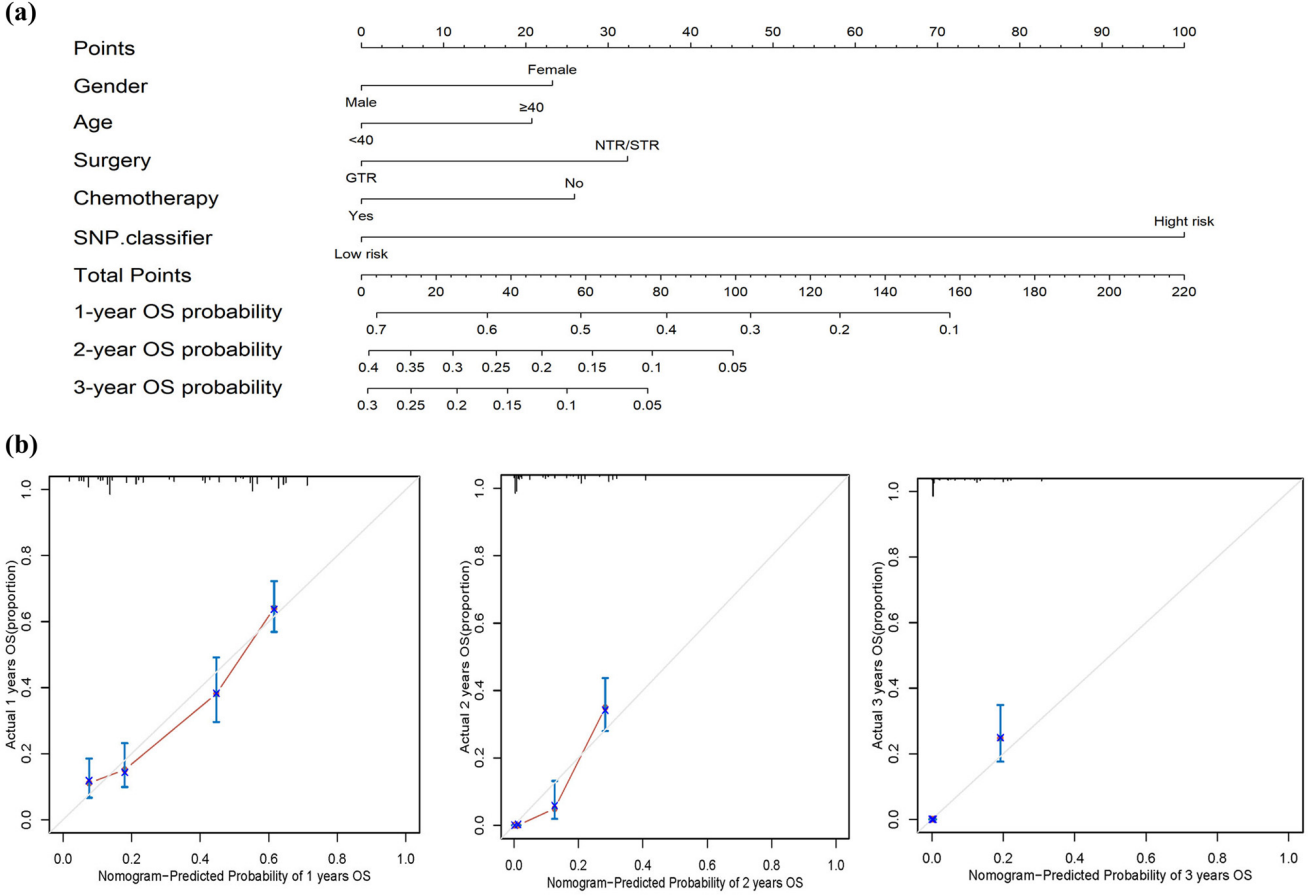
Nomogram was used to predict OS at 1, 2, and 3 years. (a) Total points and the predicted 1, 2, and 3 years OS were obtained by adding up the points of each multivariate regression variable. (b) Calibration curve. The diagonal represents the ideal fit of predicted survival and actual survival.

**Figure 7:**
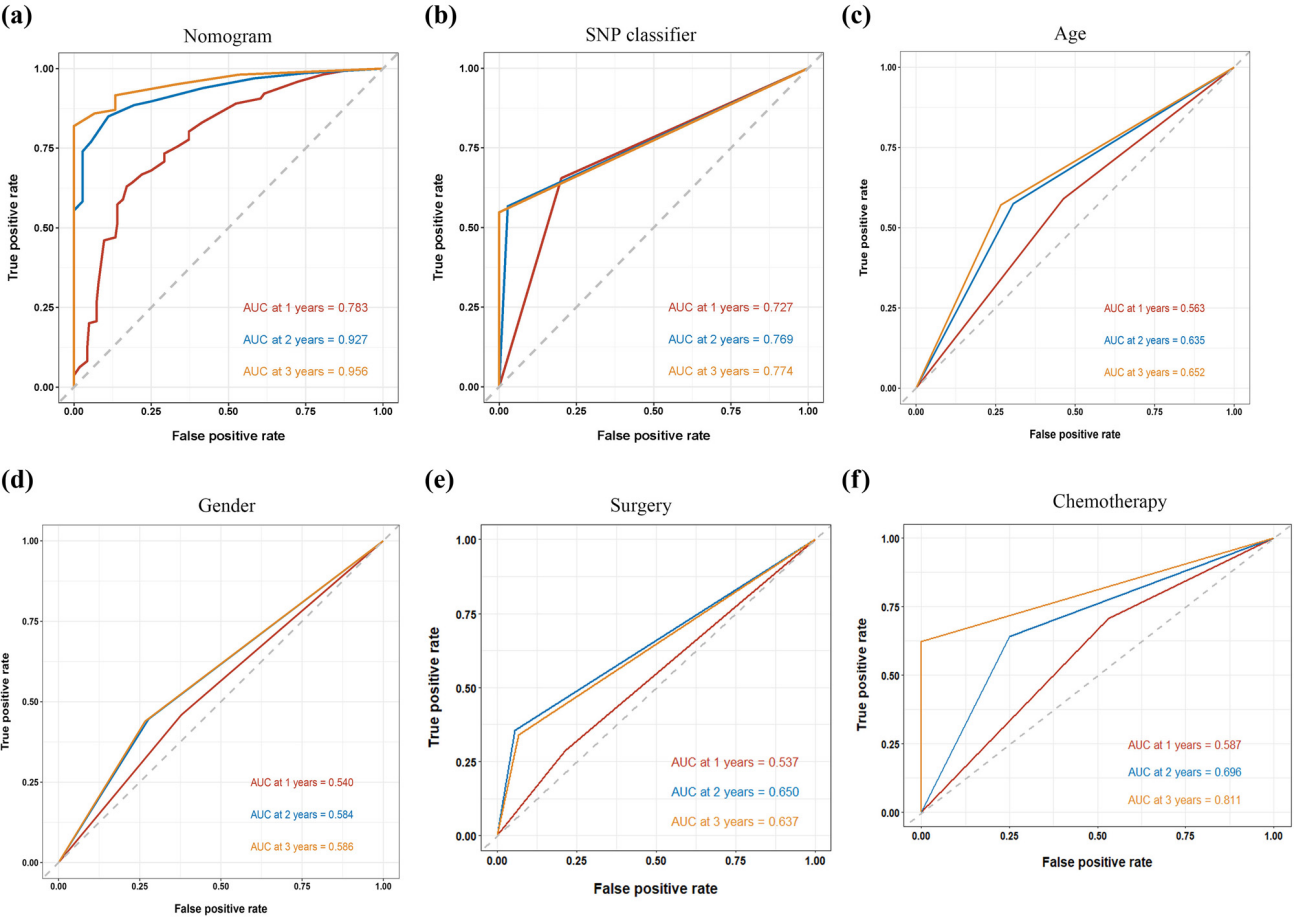
Assessment of the prognostic prediction ability of the prognostic models. (a) ROC curves for predicting the 1-year survival, 2-year survival, and 3- year survival in nomogram model comprised of SNP-based classifier and clinical features. (b) ROC curves for predicting the 1-year survival, 2-year survival, and 3- year survival in SNP-based classifier model. (c)–(f) ROC curves for predicting the 3-year survival in age, gender, surgery, and chemotherapy.

**Figure 8:**
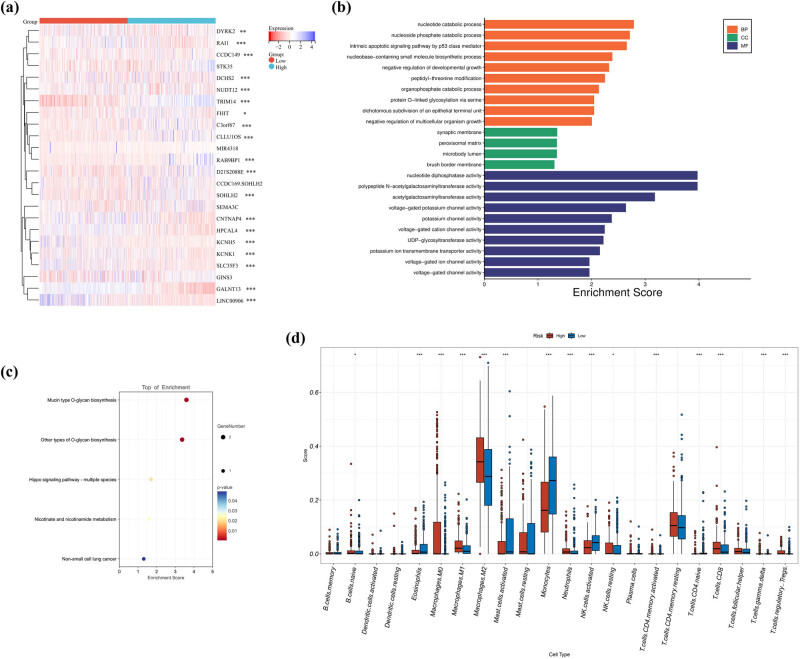
Functional and immune analysis for the two risk groups. (a) SNP-related prognostic genes heatmap in the TCGA cohort. (b) and (c) Go and KEGG analysis. (d) Enrichment scores of immune cells and immune-related pathways for the two risk groups in the TCGA cohort (* *p* < 0.05 and *** *p* < 0.001).

To facilitate the clinical application of our findings, we developed a prognostic scoring system using a nomogram based on the above results, aiming at predicting the survival time of patients at 1-year, 2-year, and 3-year. Subsequently, we identified five independent prognostic factors: age, gender, surgery, and chemotherapy. These factors were then used to construct a nomogram that estimated the OS probabilities at 1-year, 2-year, and 3-year. Figure 6a demonstrates that each prognostic parameter was assigned a score, and the sum of these scores was used to estimate the probability of 1-year, 2-year, and 3-year OS. Calibration plots were used to assess the predictive performance of the nomogram, and the results indicated a close agreement between the predicted OS and the actual 1, 2, and 3-year OS in glioma (Figure 6b). Furthermore, we constructed a ROC curve to evaluate the predictive performance of the prognostic model. As shown in Figure 7a–f, the nomogram (combining the SNP classifier with age, gender, surgery, and chemotherapy) exhibited a significantly higher predictive value for the 1-year (AUC = 0.783), 2-year (AUC = 0.927), and 3-year (AUC = 0.956) OS of patients compared to individual factors. Furthermore, we generated a heatmap to visualize the expression patterns of the 24 genes in the TCGA cohort. The results revealed significant differences in the distribution of 19 genes between the low- and high-risk subgroups (*p* < 0.05) (Figure 8a).

### 3.5 Functional analyses of the risk model

To investigate the potential biological functions and pathways associated with the RS, we conducted GO enrichment and KEGG pathway analyses using the SNPs-related prognostic genes between the low- and high-risk groups. We identified a total of 24 genes that were differentially expressed between the low-risk and high-risk groups. Specifically, seven genes were upregulated in the high-risk group, while 12 genes were downregulated. Subsequently, we conducted GO and KEGG analyses based on these genes. As shown in Figure 8b, the top three GO enrichment items in biological process (BP), cellular component, and molecular function groups were nucleotide catabolic process, nucleoside phosphate catabolic process, intrinsic apoptotic signaling pathway by p53 class mediator, synaptic membrane, peroxisomal matrix, microbody lumen, nucleotide diphosphatase activity, polypeptide *N*-acetylgalactosaminyltransferase activity, and acetylgalactosaminyltransferase activity. KEEG enrichment analysis showed that SNPs-related prognostic genes significantly enriched in mucin/other type *O*-glycan biosynthesis, Hippo signaling pathway, and nicotinate and nicotinamide metabolism (Figure 8c). The results showed that these genes were significantly enriched in immune-related pathways, such as the intrinsic apoptotic signaling pathway by p53 class mediator and Hippo signaling pathway.

### 3.6 Association of RS and the immune microenvironment

We conducted additional analyses to compare the differences in tumor‑infiltrating immune cells and immune-related pathways between the high- and low-risk populations in the TCGA cohorts (Figure 8d). The CIBERSORT analysis revealed that significantly higher ratios of eosinophils, monocytes, and gamma/delta T cells were in low-risk populations. The high-risk populations exhibited higher levels of neutrophils, macrophages (M0, M1, M2), and CD8+ T cells compared to the low-risk populations. Conversely, the high-risk populations showed significant upregulation of immune-related pathways, including naive B cells, resting of NK cells, activated CD4+ T cells memory, and T cells (tregs). In contrast, the low-risk populations exhibited upregulation of activation mast cells and naive CD4+ T cells.

## 4 Discussion

Compelling evidence suggests that molecular diagnostics have become a crucial foundation for comprehending the genetics and molecular biology of glioma, thereby aiding in the individualized treatment of glioma patients [27,28]. Recently, mounting evidence indicates the involvement of that genetic factors in glioma development, highlighting the impact of SNPs on glioma susceptibility and prognosis [29–31]. However, the prognostic value of SNPs related to prognosis in glioma has not been evaluated. In this study, we identified SNPs related to prognosis in glioma patients using GWAS analysis and constructed a prognostic model through LASSO regression analysis. Additionally, we calculated the RS using 25 SNPs, and the clinical features were found to be independent prognostic biomarkers for glioma. Furthermore, we developed a nomogram for the prognostic prediction model based on the independent prognostic factors that can predict the 1-year, 2-year, and 3-year survival rates of glioma patients. ROC curves further confirmed the superior predictive ability of the prognostic prediction model for glioma prognosis.

The RS is widely utilized for constructing significant signatures in a prognostic model [32]. In this study, we developed a prognostic model based on 25 SNPs, and glioma patients were classified into low-risk and high-risk groups by calculating the median RS derived from these SNPs. Consistent with previous findings, patients with low-RSs exhibited extended OS [33,34]. Additionally, both univariate and multivariate Cox regression analyses revealed that the SNP classifier, age, gender, surgery, and chemotherapy were independent prognostic risk factors for glioma. Nomogram are widely utilized in clinical research due to their intuitive visual presentation [35,36]. In this study, we developed a nomogram incorporating gender, age, surgery, chemotherapy, and the SNP classifier, enabling more precise prediction of individualized survival. Age and gender are risk factors for glioma incidence. The incidence of astrocytoma (including glioblastoma and diffuse or anaplastic astrocytoma) increases with age, peaking between 75 and 84 years old [37]. At all ages, the incidence of males is 40–50% higher than that of females [38]. Moreover, age and gender are considered significant factors associated with glioma prognosis [39]. The risk of prognosis increases with age, such as the poor prognosis is observed among the elderly and infirm who may not tolerate chemical or surgical treatment [40]. Females have an independent association with improved prognosis compared to males among glioma patients [41]. Additionally, surgery and chemotherapy also affect the prognosis of patients. For example, a study has demonstrated that patients with glioma who underwent surgical resection had longer survival compared to those who did not receive surgical resection [42]. Chemotherapy is a crucial adjuvant therapy, and the choice of treatment regimens and duration can impact the prognosis. Stupp et al. evaluated the efficacy of radiotherapy combined with chemotherapy in glioma treatment and observed a significant prolongation of OS time for patients [43]. SNPs are common genetic variations that can serve as indicators of genetic differences among individuals and help identify gene regions that impact prognosis. Several studies have identified SNPs associated with glioma prognosis, including variations in gene regions affecting DNA repair [22,44]. Therefore, the integration of age, gender, surgery, chemotherapy, and SNPs into the prognostic model can enhance its predictive accuracy and individualization. ROC curves were subsequently utilized to assess the diagnostic value of the nomogram for 1 (AUC = 0.783), 2 (AUC = 0.927), and 3 years (AUC = 0.956) survival rates among glioma patients. Our model exhibited significantly higher predictive accuracy compared to currently available gene-related signatures. For instance, Bingxiang et al. demonstrated a prognostic model for glioma constructed using nine prognostic-related genes that had a 3-year AUC of only 0.75 [45]. Yu et al. revealed that a prognostic model for glioma based on a 16-genes signature had an AUC of 0.86 at 3 years [46]. Additionally, Chao et al. developed a glioma prognostic model using seven pyroptosis-related genes and observed that an AUC of 0.713 at 3 years [47]. These existing prognostic models were constructed based on certain public databases and lack validation. Furthermore, our prognostic prediction model was developed based on experimental results obtained from GWAS analysis.

A glioma prognostic model for glioma was established using 25 SNPs, including rs10839 and rs9662128 in *HPCAL4*, rs10797484 in *KCNK1*/*SLC35F3*, rs1023793 in *C3orf67*/*FHIT*, rs3780471 in *TRIM14*, rs10745588 in *LOC101928617*/*CLLU1OS*, rs4133291 in *MIR4318*/*MIR924HG*, rs56407818, rs55849754, and rs7247294 in *LOC100420587*/*LINC00906*, rs62171206 in *GALNT13*, rs13126958 in *CCDC149*, rs72968021 in *DCHS2*, rs245041 in *LINC02056*, rs4235645 in *NUDT12*/*RAB9BP1*, rs17521861 in *GALNT17*, rs76970177 in *SEMA3C*/*LOC105369146*, rs7137657 in *LINC02408*/*DYRK2*, rs78518801 in *CCDC169-SOHLH2*/*SOHLH2*, rs117293024 in *KCNH5*, rs76608343 in *GINS3*, rs4888508 in *CNTNAP4*, rs12940675 in *RAI1*, rs6136889 in *PDYN-AS1*/*STK35*, and rs147068033 in *D21S2088E*/*LINC01689*. RegulomeDB is a tool for exploring annotations of variants on haplotype blocks, including candidate regulatory SNPs at disease-associated loci. According to RegulomeDB, we found evidence suggesting that these SNPs may be associated with TF binding, any motif, DNase peak, and Motif hit, potentially implicating their involvement in the regulation of these factors and their impact on glioma prognosis. Bi et al. conducted a study utilizing single-cell ATAC-seq and Hi-C to investigate the dynamic changes in chromatin organization during mouse terminal erythropoiesis [48]. They discovered that erythroid precursor cells undergo a transition from an open to a closed chromatin architecture, which coincides with the activation of erythroid genes. SNP loci might reside in chromatin structural regulatory regions, such as chromatin junction points or open regions. Nucleotide substitutions in SNPs can potentially influence the structure and function of these regions, consequently impacting gene expression regulation. This suggests a potential avenue for further exploration – whether disease-associated SNPs can also modulate disease genes by affecting 3D chromatin conformation. SNPs located in architectural regions, such as chromatin loops or anchors, have the potential to perturb structure and function due to nucleotide substitutions. Investigating the involvement of chromatin remodeling in the observed SNP associations from disease studies would be of great value.

The *HPCAL4* gene, which functions as a neural calcium sensor, has been recognized as a key molecule in glioblastoma [49]. Yu et al. discovered that *HPCAL4* could serve as a prognostic biomarker for glioblastoma [46]. Moreover, *HPCAL4* is involved in the organization and maintenance of extracellular matrix, an essential component of the tumor microenvironment that plays a crucial role in tumor growth, infiltration and metastasis, including glioma [50,51]. *KCNK1* belongs to the inwardly rectifying K(+) channel family, which helps establish the membrane potential at the K(+) balance potential [52]. By regulating cell membrane potential, *KCNK1* influences BP such as cell proliferation and apoptosis [53]. Moreover, multiple studies have demonstrated the association of *KCNK1* with the development and prognosis of various cancers, including glioma, through its regulation of tumor cell proliferation and survival [54]. *SLC35F3*, a gene associated with the nervous system, has been linked to hypertension [55] and bipolar disorder [56]. *C3orf67* is a newly discovered long noncoding RNA that plays a significant role in glioma prognosis [57]. *FHIT* functions as a tumor suppressor, and changes in its gene expression alteration are implicated in the development of glioblastoma [58]. *TRIM14* is linked to a therapeutic approach for glioma. Studies have revealed that *TRIM14* can enhance chemoresistance, malignancy, and invasion of glioma cells by modulating the Wnt/β-catenin signaling pathway [59], p38/MAPK pathway [60], and zinc finger E-box binding homeobox 2 [61]. *CLLU1OS* has been identified as a prognostic marker for prostate adenocarcinoma [62]. *GALNT13* exhibits particularly high expression in neuronal cells and may play an important role in glioma development [63]. Furthermore, a study demonstrated a strong correlation between *GALNT13* expression and poor clinical outcomes at diagnosis in human neuroblastoma [64]. *CCDC149* has recently been identified as a genetic susceptibility gene for thyroid carcinoma [65]. *DCHS2* may contribute to the occurrence of Alzheimer’s disease [66] and colorectal cancer [67]. Additionally, An et al. demonstrated that the unconventional *DCHS2* gene carries frameshift mutations that could deactivate cell adhesion-related functions, potentially serving as a characteristic of gastric and colorectal cancers with high microsatellite instability [68]. *NUDT12*, which is involved in cellular energetics, serves as a critical prognostic biomarker in glioma progression [69]. *GALNT17* encodes the *N*-acetylgalactosaminyltransferase protein and plays a significant role in cell signaling, neurite outgrowth, neurotransmitter activity, and neurite sensing. A recent study has suggested the involvement of *GALNT17* in brain development [70]. *SEMA3C* is closely related to glioma progression, and several studies have demonstrated that its high expression promotes glioma malignancy and is indicative of a poor prognosis [71,72]. *LINC02408* serves as a prognostic signature for ovarian cancer [73] and breast cancer [74], as well as a diagnostic marker for thyroid cancer [75]. *DYRK2* is expressed in human tumors and can suppress glioma cell migration, affecting E-cadherin and vimentin expression in the PI3K/AKT/GSK3β signaling pathway. It has the potential to be a therapeutic target [76]. *KCNH5* is widely expressed in the human brain, and mutations in this gene contribute to abnormal brain development, potentially playing a role in glioma development [77]. *GINS3* exhibits high expression in numerous tumor tissues, including glioma, and is associated with the development and prognosis of human cancers [78–80]. *CNTNAP4* may serve as a prognostic biomarker for hepatocellular carcinoma [81], prostate cancer [82], and breast cancer [83]. *RAI1* may counteract viral infection by regulating immune reactions in astrocytoma cells [84]. *STK35* can influence the chemoresistance of colorectal cancer by promoting glycolysis and inhibiting apoptosis through the regulation of the AKT pathway [85]. Additionally, it was observed that STK35 modulates apoptosis and proliferation in osteosarcoma [86]. In summary, these genes are closely related to the occurrence and prognosis of glioma through their influence on specific BPs. Consequently, constructing glioma prognostic models using prognosis-related SNPs holds significant biological significance and potential effectiveness.

Functional analyses revealed significant enrichment of these genes in immune-related pathways, including the intrinsic apoptotic signaling pathway by p53 class mediator and the Hippo signaling pathway. Additionally, we examined the disparities in immune-related pathways and immune cell infiltration between the high- and low-risk groups. Our study suggests that the immune microenvironment may play a role in enhancing glioma prognosis, but further verification is required.

Our study has several limitations. First, our prognostic prediction model is derived from the Han population and requires validation in other ethnic populations. Second, the molecular mechanism underlying the association between the 25 key SNPs and glioma prognosis are still unclear, necessitating further molecular experiments to elucidate the biological mechanisms. Despite these limitations, our study is the first to develop a prognostic model for glioma based on GWAS-identified SNPs, offering significant value in enhancing prognosis and guiding targeted therapy.

In conclusion, we identified 25 SNPs significantly associated with glioma prognosis. Moreover, our study demonstrated that the SNP classifier, along with age, gender, surgery, and chemotherapy, serve as independent prognostic factors for glioma. Subsequently, we developed a nomogram using these independent prognostic factors to predict the 1-year, 2-year, and 3-year survival rates of glioma patients. This model will assist clinicians in accurately evaluating patient prognosis and guide follow-up and treatment decisions.

## Supplementary Material

supplementary material
