# Supplementary material for: The construction of a novel prognostic prediction model for glioma based on GWAS-identified prognostic-related risk loci
Source: Open Med (Wars). 2024 Mar 15;19(1):20240895. doi: 10.1515/med-2024-0895 (PMC10996933; doi:10.1515/med-2024-0895)
Supplement: supplementary material [file med-2024-0895-sm.pdf]

Supplementary material

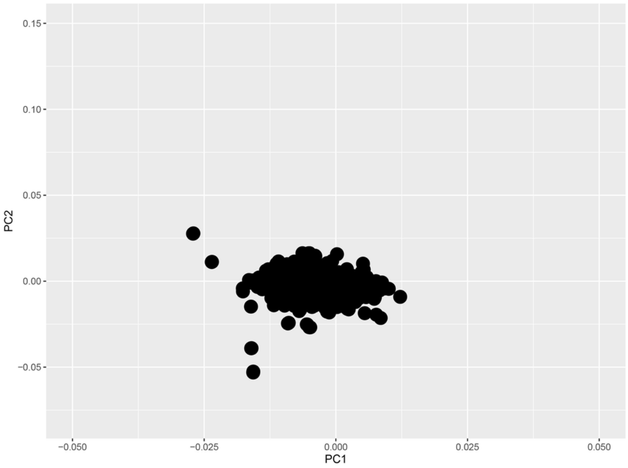

Figure S1: PCA analysis of the study population, PCA: principal component analysis.

Table S1: Candidate SNPs for OS risk in glioma

SNP	Chr	BP	Gene	HR (95% CI)	*p*
rs10839	1	39679146	*HPCAL4*	0.73 (0.63–0.85)	4.26×10^{-5}
rs113459798	1	39684203	*HPCAL4*	0.73 (0.62–0.85)	4.48×10^{-5}
rs9662128	1	39684484	*HPCAL4*	0.72 (0.62–0.84)	3.56×10^{-5}
rs10797484	1	233791256	*KCNK1*; *SLC35F3*	0.74 (0.64–0.85)	3.66×10^{-5}
rs76015569	2	16861589	*FAM49A*; *RAD51AP2*	1.87 (1.41–2.50)	1.70×10^{-5}
rs62171206	2	154257864	*GALNT13*	1.77 (1.37–2.29)	1.15×10^{-5}
rs62171251	2	154261639	*GALNT13*	1.73 (1.33–2.24)	4.04×10^{-5}
rs10929964	2	160031171	*PLA2R1*	0.72 (0.61–0.84)	4.58×10^{-5}
rs1023793	3	59341735	*C3orf67*; *FHIT*	0.72 (0.62–0.83)	1.55×10^{-5}
rs13126958	4	24976807	*CCDC149*	1.48 (1.26–1.74)	1.87×10^{-6}
rs7683047	4	24977741	*CCDC149*	1.4 (1.20–1.63)	2.66×10^{-5}
rs72968021	4	154373450	*DCHS2*	2.11 (1.57–2.84)	7.43×10^{-7}
rs903084	5	4104132	*IRX1*; *LINC02114*	1.33 (1.16–1.52)	3.26×10^{-5}
rs923840	5	4104571	*IRX1*; *LINC02114*	1.33 (1.16–1.52)	3.26×10^{-5}
rs12519357	5	4107935	*IRX1*; *LINC02114*	1.34 (1.17–1.53)	1.97×10^{-5}
rs245041	5	72640578	*LINC02056*	1.33 (1.16–1.51)	3.33×10^{-5}
rs4235645	5	104799715	*NUDT12*; *RAB9BP1*	1.38 (1.18–1.62)	4.90×10^{-5}
rs2652251	5	123982950	*CSNK1G3*; *LINC01170*	1.48 (1.24–1.76)	1.69×10^{-5}
rs34405476	6	149171541	*UST*; *TAB2*	1.89 (1.39–2.58)	4.99×10^{-5}
rs2867046	7	71238910	*GALNT17*	1.46 (1.22–1.74)	2.94×10^{-5}
rs17521861	7	71244666	*GALNT17*	1.5 (1.26–1.79)	5.52×10^{-6}
rs62468562	7	78512265	*MAGI2*	1.8 (1.37–2.36)	2.49×10^{-5}
rs62468563	7	78512614	*MAGI2*	1.8 (1.37–2.36)	2.49×10^{-5}
rs3801362	7	78517908	*MAGI2*	1.78 (1.36–2.34)	3.14×10^{-5}
rs74519761	7	81363334	*SEMA3C*; *LOC105369146*	1.47 (1.23–1.76)	2.94×10^{-5}
rs76970177	7	81364751	*SEMA3C*; *LOC105369146*	1.48 (1.24–1.77)	1.42×10^{-5}
rs79617578	7	81376899	*SEMA3C*; *LOC105369146*	1.44 (1.21–1.72)	4.93×10^{-5}
rs72508697	7	81377136	*SEMA3C*; *LOC105369146*	1.44 (1.21–1.72)	4.93×10^{-5}
rs73492791	9	98071643	*NANS*	0.65 (0.53–0.80)	2.72×10^{-5}
rs3780471	9	98085269	*TRIM14*	0.65 (0.54–0.79)	2.09×10^{-5}
rs2249304	10	8226118	*GATA3*; *LINC00708*	1.5 (1.25–1.79)	1.40×10^{-5}
rs2249305	10	8226144	*GATA3*; *LINC00708*	1.5 (1.25–1.79)	1.40×10^{-5}
rs7137657	12	67605696	*LINC02408*; *DYRK2*	1.68 (1.32–2.12)	1.79×10^{-5}
rs1579655	12	92242372	*LOC101928617*; *CLLU1OS*	0.72 (0.61–0.84)	3.25×10^{-5}
rs1866248	12	92242857	*LOC101928617*; *CLLU1OS*	0.72 (0.61–0.84)	3.25×10^{-5}
rs7303321	12	92246410	*LOC101928617*; *CLLU1OS*	0.72 (0.62–0.84)	3.61×10^{-5}
rs10859287	12	92248968	*LOC101928617*; *CLLU1OS*	0.7 (0.59–0.82)	7.52×10^{-6}
rs10745588	12	92250325	*LOC101928617*; *CLLU1OS*	0.69 (0.59–0.81)	6.83×10^{-6}
rs872969	12	92252224	*LOC101928617*; *CLLU1OS*	0.71 (0.60–0.83)	1.60×10^{-5}
rs10777386	12	92253150	*LOC101928617*; *CLLU1OS*	0.71 (0.60–0.83)	1.60×10^{-5}
rs2805890	12	92254617	*LOC101928617*; *CLLU1OS*	0.71 (0.60–0.83)	1.60×10^{-5}

(Continued)

Table S1: *Continued*

SNP	Chr	BP	Gene	HR (95% CI)	*p*
rs1545152	12	92261661	*LOC101928617*; *CLLU1OS*	0.71 (0.61–0.83)	2.37×10^{-5}
rs6538323	12	92264008	*LOC101928617*; *CLLU1OS*	0.71 (0.61–0.83)	2.37×10^{-5}
rs6538324	12	92264747	*LOC101928617*; *CLLU1OS*	0.71 (0.61–0.83)	2.37×10^{-5}
rs11106481	12	92265670	*LOC101928617*; *CLLU1OS*	0.71 (0.61–0.83)	2.37×10^{-5}
rs28421429	12	132346755	*LOC101928416*; *FBRSL1*	0.74 (0.64–0.85)	3.17×10^{-5}
rs78351393	13	36170257	*CCDC169-SOHLH2*; *SOHLH2*	1.78 (1.37–2.32)	1.94×10^{-5}
rs76601148	13	36171919	*CCDC169-SOHLH2*; *SOHLH2*	1.78 (1.37–2.32)	1.94×10^{-5}
rs78518801	13	36188063	*CCDC169-SOHLH2*; *SOHLH2*	1.86 (1.43–2.41)	2.94×10^{-6}
rs79882008	13	36197158	*CCDC169-SOHLH2*; *SOHLH2*	1.82 (1.40–2.35)	5.24×10^{-6}
rs2322895	13	36198931	*CCDC169-SOHLH2*; *SOHLH2*	1.82 (1.41–2.35)	3.88×10^{-6}
rs78972162	13	36199197	*CCDC169-SOHLH2*; *SOHLH2*	1.82 (1.41–2.35)	3.88×10^{-6}
rs2149425	13	36201154	*CCDC169-SOHLH2*; *SOHLH2*	1.79 (1.39–2.31)	6.89×10^{-6}
rs17053630	13	36202364	*CCDC169-SOHLH2*; *SOHLH2*	1.79 (1.39–2.31)	6.89×10^{-6}
rs75933854	13	36205120	*CCDC169-SOHLH2*; *SOHLH2*	1.72 (1.34–2.20)	2.16×10^{-5}
rs77706449	13	36206125	*CCDC169-SOHLH2*; *SOHLH2*	1.78 (1.38–2.30)	7.95×10^{-6}
rs74694612	13	36209455	*CCDC169-SOHLH2*; *SOHLH2*	1.82 (1.41–2.34)	4.48×10^{-6}
rs113710221	13	94213059	*GPC6*	1.52 (1.24–1.86)	4.88×10^{-5}
rs117293024	14	62952042	*KCNH5*	1.77 (1.36–2.32)	2.87×10^{-5}
rs4906709	15	24685113	*NPAP1*; *SNRPN*	1.36 (1.17–1.57)	4.57×10^{-5}
rs12905620	15	24685231	*NPAP1*; *SNRPN*	1.36 (1.17–1.57)	4.53×10^{-5}
rs12595070	15	24686364	*NPAP1*; *SNRPN*	1.37 (1.18–1.58)	2.94×10^{-5}
rs12917107	15	24688734	*NPAP1*; *SNRPN*	1.36 (1.18–1.58)	3.92×10^{-5}
rs12899531	15	24688754	*NPAP1*; *SNRPN*	1.36 (1.18–1.58)	3.92×10^{-5}
rs12899547	15	24688793	*NPAP1*; *SNRPN*	1.36 (1.18–1.58)	3.92×10^{-5}
rs12899555	15	24688797	*NPAP1*; *SNRPN*	1.36 (1.18–1.58)	3.92×10^{-5}
rs12899317	15	24689014	*NPAP1*; *SNRPN*	1.36 (1.18–1.57)	3.73×10^{-5}
rs35645715	15	24689231	*NPAP1*; *SNRPN*	1.37 (1.18–1.60)	3.34×10^{-5}
rs2883645	15	24718228	*NPAP1*; *SNRPN*	1.37 (1.18–1.58)	2.64×10^{-5}
rs17681323	15	29047006	*APBA2*	1.95 (1.45–2.62)	8.54×10^{-6}
rs76608343	16	58395747	*GINS3*	1.37 (1.18–1.58)	2.70×10^{-5}
rs9928692	16	76430774	*CNTNAP4*	1.34 (1.17–1.53)	3.02×10^{-5}
rs4888508	16	76438786	*CNTNAP4*	1.36 (1.18–1.56)	1.58×10^{-5}
rs13331860	16	76442572	*CNTNAP4*	1.35 (1.17–1.55)	2.55×10^{-5}
rs4925108	17	17746109	*RAI1*	1.73 (1.33–2.25)	4.75×10^{-5}
rs12940675	17	17747900	*RAI1*	1.76 (1.36–2.28)	1.96×10^{-5}
rs12945601	17	17750097	*RAI1*	1.76 (1.36–2.28)	1.96×10^{-5}
rs55767800	17	17771653	*RAI1*	1.73 (1.34–2.22)	2.14×10^{-5}
rs12950737	17	17775259	*RAI1*	1.73 (1.34–2.22)	2.14×10^{-5}
rs941446	17	17776959	*SMCR5*	1.86 (1.42–2.42)	5.94×10^{-6}
rs12940418	17	17782738	*RAI1*	1.74 (1.33–2.27)	4.83×10^{-5}

(Continued)

Table S1: *Continued*

SNP	Chr	BP	Gene	HR (95% CI)	*p*
rs12952495	17	17784462	*RAI1*	1.75 (1.34–2.28)	3.55×10^{-5}
rs75402227	17	57411287	*MSI2*	1.63 (1.3–2.05)	2.83×10^{-5}
rs7218689	17	57413371	*MSI2*	1.63 (1.30–2.05)	2.83×10^{-5}
rs8071635	17	57415791	*MSI2*	1.63 (1.30–2.05)	2.66×10^{-5}
rs17761485	17	57416799	*MSI2*	1.63 (1.30–2.05)	2.66×10^{-5}
rs9892791	17	57417734	*MSI2*	1.63 (1.30–2.05)	2.59×10^{-5}
rs17834199	17	57418398	*MSI2*	1.63 (1.30–2.05)	2.90×10^{-5}
rs7213277	17	57420716	*MSI2*	1.66 (1.31–2.10)	2.91×10^{-5}
rs61377283	18	38870239	*MIR4318*; *MIR924HG*	0.76 (0.66–0.87)	4.63×10^{-5}
rs11876568	18	38870468	*MIR4318*; *MIR924HG*	0.76 (0.66–0.87)	4.63×10^{-5}
rs4133291	18	38898280	*MIR4318*; *MIR924HG*	0.75 (0.66–0.86)	2.62×10^{-5}
rs56407818	19	28788472	*LOC100420587*; *LINC00906*	0.65 (0.53–0.79)	2.30×10^{-5}
rs55849754	19	28788758	*LOC100420587*; *LINC00906*	0.65 (0.53–0.79)	2.30×10^{-5}
rs17498203	19	28788827	*LOC100420587*; *LINC00906*	0.65 (0.53–0.79)	2.30×10^{-5}
rs7247294	19	28789022	*LOC100420587*; *LINC00906*	0.65 (0.54–0.79)	2.08×10^{-5}
rs12151001	19	28792479	*LOC100420587*; *LINC00906*	0.65 (0.53–0.79)	2.30×10^{-5}
rs73029061	19	28793073	*LOC100420587*; *LINC00906*	0.65 (0.53–0.79)	2.30×10^{-5}
rs6035474	20	2061818	*PDYN-AS1*; *STK35*	1.67 (1.32–2.11)	1.54×10^{-5}
rs6136889	20	2064746	*PDYN-AS1*; *STK35*	1.69 (1.35–2.13)	6.64×10^{-6}
rs6112683	20	2066802	*PDYN-AS1*; *STK35*	1.61 (1.28–2.03)	4.51×10^{-5}
rs6112685	20	2066820	*PDYN-AS1*; *STK35*	1.61 (1.28–2.03)	4.51×10^{-5}
rs6112686	20	2066833	*PDYN-AS1*; *STK35*	1.61 (1.28–2.03)	4.51×10^{-5}
rs6136898	20	2067172	*PDYN-AS1*; *STK35*	1.62 (1.29–2.02)	2.93×10^{-5}
rs2295515	20	5471173	*LOC643406*	0.63 (0.51–0.79)	3.56×10^{-5}
rs147068033	21	23637454	*D21S2088E*; *LINC01689*	1.37 (1.19–1.59)	1.94×10^{-5}

SNP: single nucleotide polymorphism, Chr: chromosome, BP: biological position, OS: overall survival, HR: hazard ratio, CI: confidence interval.

Table S2: Candidate SNPs for PFS risk in glioma

SNP	Chr	BP	Gene	HR (95% CI)	*p*
rs784628	1	39678418	*HPCAL4*	0.72 (0.62–0.84)	3.59×10^{-5}
rs10839	1	39679146	*HPCAL4*	0.71 (0.61–0.83)	1.16×10^{-5}
rs113459798	1	39684203	*HPCAL4*	0.71 (0.61–0.83)	1.12×10^{-5}
rs9662128	1	39684484	*HPCAL4*	0.7 (0.60–0.82)	8.64×10^{-6}
rs6587963	1	62258282	*KANK4*	0.74 (0.64–0.85)	3.30×10^{-5}
rs1543313	1	166874622	*TADA1*	0.74 (0.64–0.85)	1.61×10^{-5}
rs2072740	1	166875791	*TADA1*	0.74 (0.64–0.85)	1.68×10^{-5}
rs9633326	1	166884383	*TADA1*; *ILDR2*	0.75 (0.66–0.86)	4.38×10^{-5}
rs10800276	1	166889704	*TADA1*; *ILDR2*	0.75 (0.66–0.86)	4.74×10^{-5}
rs10797484	1	233791256	*KCNK1*; *SLC35F3*	0.73 (0.63–0.84)	2.18×10^{-5}
rs34073169	2	132409911	*FAM201B*; *GPR39*	1.81 (1.36–2.41)	4.96×10^{-5}
rs62171206	2	154257864	*GALNT13*	1.72 (1.33–2.22)	3.01×10^{-5}
rs1023793	3	59341735	*C3orf67*; *FHIT*	0.73 (0.62–0.84)	3.10×10^{-5}
rs13126958	4	24976807	*CCDC149*	1.47 (1.25–1.74)	3.35×10^{-6}
rs7683047	4	24977741	*CCDC149*	1.39 (1.19–1.63)	3.61×10^{-5}
rs56981143	4	74392511	*EREG*; *AREG*	1.86 (1.39–2.48)	2.32×10^{-5}
rs62315988	4	87635119	*DSPP*; *DMP1*	1.32 (1.15–1.51)	5.00×10^{-5}
rs59169242	4	87635582	*DSPP*; *DMP1*	1.32 (1.15–1.51)	5.00×10^{-5}
rs990772	4	87670841	*DMP1*; *IBSP*	1.32 (1.16–1.51)	4.66×10^{-5}
rs6841293	4	87672470	*DMP1*; *IBSP*	1.32 (1.16–1.51)	4.70×10^{-5}
rs72968021	4	154373450	*DCHS2*	2 (1.49–2.69)	3.82×10^{-6}
rs10017158	4	163178515	*NAF1*; *NPY1R*	1.85 (1.39–2.47)	2.41×10^{-5}
rs6536707	4	163201874	*NAF1*; *NPY1R*	1.9 (1.43–2.51)	8.25×10^{-6}
rs2874239	4	163204891	*NAF1*; *NPY1R*	1.88 (1.42–2.50)	1.23×10^{-5}
rs62335074	4	163215246	*NAF1*; *NPY1R*	1.83 (1.38–2.43)	2.46×10^{-5}
rs245041	5	72640578	*LINC02056*	1.32 (1.16–1.52)	3.94×10^{-5}
rs183734784	5	96362102	*LOC101929710*	1.86 (1.38–2.50)	3.97×10^{-5}
rs4235645	5	104799715	*NUDT12*; *RAB9BP1*	1.4 (1.20–1.64)	2.86×10^{-5}
rs58982	5	104824432	*NUDT12*; *RAB9BP1*	1.4 (1.19–1.64)	2.90×10^{-5}
rs17521861	7	71244666	*GALNT17*	1.48 (1.24–1.77)	1.41×10^{-5}
rs74519761	7	81363334	*SEMA3C*; *LOC105369146*	1.48 (1.24–1.78)	1.92×10^{-5}
rs76970177	7	81364751	*SEMA3C*; *LOC105369146*	1.49 (1.25–1.78)	9.78×10^{-6}
rs79617578	7	81376899	*SEMA3C*; *LOC105369146*	1.45 (1.21–1.73)	4.20×10^{-5}
rs72508697	7	81377136	*SEMA3C*; *LOC105369146*	1.45 (1.21–1.73)	4.20×10^{-5}
rs4742272	9	6946719	*KDM4C*	1.78 (1.37–2.32)	1.39×10^{-5}
rs28632803	9	6949133	*KDM4C*	1.7 (1.32–2.20)	4.34×10^{-5}
rs12345802	9	6949624	*KDM4C*	1.7 (1.32–2.20)	4.34×10^{-5}
rs74573409	9	6950922	*KDM4C*	1.7 (1.32–2.19)	3.28×10^{-5}
rs10975931	9	6951639	*KDM4C*	1.7 (1.32–2.19)	3.28×10^{-5}
rs10975932	9	6951700	*KDM4C*	1.7 (1.32–2.19)	3.28×10^{-5}
rs7868655	9	6951869	*KDM4C*	1.7 (1.32–2.19)	3.28×10^{-5}

(Continued)

Table S2: *Continued*

SNP	Chr	BP	Gene	HR (95% CI)	*p*
rs28453377	9	6954040	*KDM4C*	1.7 (1.32–2.19)	3.28×10^{-5}
rs13440337	9	6954457	*KDM4C*	1.7 (1.32–2.18)	3.69×10^{-5}
rs13440172	9	6954537	*KDM4C*	1.7 (1.32–2.18)	3.69×10^{-5}
rs12346482	9	6955015	*KDM4C*	1.7 (1.32–2.18)	3.38×10^{-5}
rs10124531	9	6955476	*KDM4C*	1.7 (1.32–2.18)	3.69×10^{-5}
rs12335500	9	6957024	*KDM4C*	1.7 (1.32–2.18)	3.69×10^{-5}
rs12335507	9	6957061	*KDM4C*	1.7 (1.32–2.18)	3.69×10^{-5}
rs10967222	9	26195521	*LOC100506422*; *CAAP1*	1.34 (1.17–1.53)	2.38×10^{-5}
rs2840293	9	26196598	*LOC100506422*; *CAAP1*	1.33 (1.16–1.52)	3.04×10^{-5}
rs10812365	9	26198083	*LOC100506422*; *CAAP1*	1.33 (1.16–1.52)	3.04×10^{-5}
rs73492791	9	98071643	*NANS*	0.65 (0.53–0.80)	3.13×10^{-5}
rs3780471	9	98085269	*TRIM14*	0.65 (0.53–0.79)	1.59×10^{-5}
rs2071151	11	77159412	*MYO7A*	1.36 (1.17–1.57)	4.05×10^{-5}
rs7137657	12	67605696	*LINC02408*; *DYRK2*	1.69 (1.33–2.15)	1.97×10^{-5}
rs1579655	12	92242372	*LOC101928617*; *CLLU1OS*	0.71 (0.61–0.84)	2.51×10^{-5}
rs1866248	12	92242857	*LOC101928617*; *CLLU1OS*	0.71 (0.61–0.84)	2.51×10^{-5}
rs7303321	12	92246410	*LOC101928617*; *CLLU1OS*	0.72 (0.61–0.84)	3.06×10^{-5}
rs10859287	12	92248968	*LOC101928617*; *CLLU1OS*	0.69 (0.59–0.81)	5.72×10^{-6}
rs10745588	12	92250325	*LOC101928617*; *CLLU1OS*	0.69 (0.59–0.81)	5.26×10^{-6}
rs872969	12	92252224	*LOC101928617*; *CLLU1OS*	0.7 (0.60–0.82)	1.23×10^{-5}
rs10777386	12	92253150	*LOC101928617*; *CLLU1OS*	0.7 (0.60–0.82)	1.23×10^{-5}
rs2805890	12	92254617	*LOC101928617*; *CLLU1OS*	0.7 (0.60–0.82)	1.23×10^{-5}
rs1545152	12	92261661	*LOC101928617*; *CLLU1OS*	0.71 (0.60–0.83)	1.65×10^{-5}
rs6538323	12	92264008	*LOC101928617*; *CLLU1OS*	0.71 (0.60–0.83)	1.65×10^{-5}
rs6538324	12	92264747	*LOC101928617*; *CLLU1OS*	0.71 (0.60–0.83)	1.65×10^{-5}
rs11106481	12	92265670	*LOC101928617*; *CLLU1OS*	0.71 (0.60–0.83)	1.65×10^{-5}
rs12828044	12	95440335	*MIR3685*; *METAP2*	1.44 (1.21–1.71)	3.37×10^{-5}
rs78518801	13	36188063	*CCDC169-SOHLH2*; *SOHLH2*	1.8 (1.38–2.34)	1.34×10^{-5}
rs79882008	13	36197158	*CCDC169-SOHLH2*; *SOHLH2*	1.72 (1.32–2.22)	4.84×10^{-5}
rs2322895	13	36198931	*CCDC169-SOHLH2*; *SOHLH2*	1.73 (1.33–2.24)	3.52×10^{-5}
rs78972162	13	36199197	*CCDC169-SOHLH2*; *SOHLH2*	1.73 (1.33–2.24)	3.52×10^{-5}
rs2149425	13	36201154	*CCDC169-SOHLH2*; *SOHLH2*	1.71 (1.32–2.21)	4.26×10^{-5}
rs17053630	13	36202364	*CCDC169-SOHLH2*; *SOHLH2*	1.71 (1.32–2.21)	4.26×10^{-5}
rs77706449	13	36206125	*CCDC169-SOHLH2*; *SOHLH2*	1.7 (1.32–2.20)	4.85×10^{-5}
rs74694612	13	36209455	*CCDC169-SOHLH2*; *SOHLH2*	1.72 (1.33–2.23)	4.00×10^{-5}
rs67900844	14	40666202	*FBXO33*; *LINC02315*	1.36 (1.18–1.56)	1.80×10^{-5}
rs11157158	14	40669757	*FBXO33*; *LINC02315*	1.35 (1.17–1.55)	2.94×10^{-5}
rs117293024	14	62952042	*KCNH5*	1.78 (1.35–2.35)	4.66×10^{-5}
rs112657491	15	40255096	*BUB1B–PAK6*; *PAK6*	1.85 (1.39–2.47)	2.59×10^{-5}
rs2098079	16	5986344	*MIR8065*; *RBFOX1*	1.79 (1.40–2.30)	3.38×10^{-6}

(Continued)

Table S2: *Continued*

SNP	Chr	BP	Gene	HR (95% CI)	*p*
rs76608343	16	58395747	*GINS3*	1.36 (1.18–1.57)	3.28×10^{-5}
rs4888508	16	76438786	*CNTNAP4*	1.33 (1.16–1.53)	4.79×10^{-5}
rs12940675	17	17747900	*RAI1*	1.76 (1.35–2.30)	3.06×10^{-5}
rs12945601	17	17750097	*RAI1*	1.76 (1.35–2.30)	3.06×10^{-5}
rs941446	17	17776959	*SMCR5*	1.8 (1.37–2.36)	2.20×10^{-5}
rs61377283	18	38870239	*MIR4318*; *MIR924HG*	0.75 (0.65–0.85)	2.04×10^{-5}
rs11876568	18	38870468	*MIR4318*; *MIR924HG*	0.75 (0.65–0.85)	2.04×10^{-5}
rs4133291	18	38898280	*MIR4318*; *MIR924HG*	0.73 (0.64–0.84)	8.18×10^{-6}
rs56407818	19	28788472	*LOC100420587*; *LINC00906*	0.65 (0.53–0.80)	3.12×10^{-5}
rs55849754	19	28788758	*LOC100420587*; *LINC00906*	0.65 (0.53–0.80)	3.12×10^{-5}
rs17498203	19	28788827	*LOC100420587*; *LINC00906*	0.65 (0.53–0.80)	3.12×10^{-5}
rs7247294	19	28789022	*LOC100420587*; *LINC00906*	0.66 (0.54–0.80)	2.75×10^{-5}
rs12151001	19	28792479	*LOC100420587*; *LINC00906*	0.65 (0.53–0.80)	3.12×10^{-5}
rs73029061	19	28793073	*LOC100420587*; *LINC00906*	0.65 (0.53–0.80)	3.12×10^{-5}
rs6035474	20	2061818	*PDYN–AS1*; *STK35*	1.63 (1.29–2.06)	4.51×10^{-5}
rs6136889	20	2064746	*PDYN–AS1*; *STK35*	1.66 (1.32–2.09)	1.71×10^{-5}
rs2424343	20	20307846	*CFAP61*	0.72 (0.62–0.84)	2.36×10^{-5}
rs2424346	20	20308725	*CFAP61*	0.72 (0.62–0.84)	2.36×10^{-5}
rs2424347	20	20309880	*CFAP61*	0.72 (0.62–0.84)	1.52×10^{-5}
rs2424348	20	20310097	*CFAP61*	0.72 (0.62–0.84)	1.52×10^{-5}
rs2145057	20	20310341	*CFAP61*	0.72 (0.62–0.84)	1.52×10^{-5}
rs6035602	20	20311542	*CFAP61*	0.72 (0.62–0.84)	1.52×10^{-5}
rs73118471	20	20318426	*CFAP61*	0.72 (0.62–0.83)	1.43×10^{-5}
rs6136992	20	20320706	*CFAP61*	0.72 (0.62–0.83)	1.34×10^{-5}
rs913393	20	20321240	*CFAP61*	0.72 (0.62–0.83)	1.17×10^{-5}
rs73118488	20	20325346	*CFAP61*	0.72 (0.62–0.83)	1.26×10^{-5}
rs6075644	20	20326120	*CFAP61*	0.72 (0.62–0.83)	1.26×10^{-5}
rs6046799	20	20330048	*CFAP61*	0.72 (0.62–0.84)	1.95×10^{-5}
rs6081963	20	20332771	*CFAP61*	0.72 (0.62–0.84)	2.04×10^{-5}
rs6046801	20	20333521	*CFAP61*	0.73 (0.63–0.84)	2.22×10^{-5}
rs6046802	20	20334991	*CFAP61*	0.73 (0.63–0.84)	2.04×10^{-5}
rs728415	20	20335635	*CFAP61*	0.72 (0.62–0.84)	1.34×10^{-5}
rs3092130	20	43102562	*PTPRT*	1.46 (1.22–1.74)	4.40×10^{-5}
rs147068033	21	23637454	*D21S2088E*; *LINC01689*	1.37 (1.19–1.59)	1.87×10^{-5}

SNP: single nucleotide polymorphism, Chr: chromosome, BP: biological position, PFS: progression–free survival, HR: hazard ratio, CI: confidence interval.

Table S3: The association of genetic loci with OS and PFS risk in glioma

SNPs	Gene	Chr	BP	OS		PFS	
				HR (95% CI)	*p*	HR (95% CI)	*p*
rs10839	*HPCAL4*	1	39679146	0.73 (0.63–0.85)	4.26×10^{-5}	0.71 (0.61–0.83)	1.16×10^{-5}
rs113459798	*HPCAL4*	1	39684203	0.73 (0.62–0.85)	4.48×10^{-5}	0.71 (0.61–0.83)	1.12×10^{-5}
rs9662128	*HPCAL4*	1	39684484	0.72 (0.62–0.84)	3.56×10^{-5}	0.7 (0.60–0.82)	8.64×10^{-6}
rs10797484	*KCNK1*; *SLC35F3*	1	233791256	0.74 (0.64–0.85)	3.66×10^{-5}	0.73 (0.63–0.84)	2.18×10^{-5}
rs62171206	*GALNT13*	2	154257864	1.77 (1.37–2.29)	1.15×10^{-5}	1.72 (1.33–2.22)	3.01×10^{-5}
rs1023793	*C3orf67*; *FHIT*	3	59341735	0.72 (0.62–0.83)	1.55×10^{-5}	0.73 (0.62–0.84)	3.10×10^{-5}
rs13126958	*CCDC149*	4	24976807	1.48 (1.26–1.74)	1.87×10^{-6}	1.47 (1.25–1.74)	3.35×10^{-6}
rs7683047	*CCDC149*	4	24977741	1.4 (1.20–1.63)	2.66×10^{-5}	1.39 (1.19–1.63)	3.61×10^{-5}
rs72968021	*DCHS2*	4	154373450	2.11 (1.57–2.84)	7.43×10^{-6}	2 (1.49–2.69)	3.82×10^{-6}
rs245041	*LINC02056*	5	72640578	1.33 (1.16–1.51)	3.33×10^{-5}	1.32 (1.16–1.52)	3.94×10^{-5}
rs4235645	*NUDT12*; *RAB9BP1*	5	104799715	1.38 (1.18–1.62)	4.90×10^{-5}	1.4 (1.20–1.64)	2.86×10^{-5}
rs17521861	*GALNT17*	7	71244666	1.5 (1.26–1.79)	5.52×10^{-6}	1.48 (1.24–1.77)	1.41×10^{-5}
rs74519761	*SEMA3C*; *LOC105369146*	7	81363334	1.47 (1.23–1.76)	2.94×10^{-5}	1.48 (1.24–1.78)	1.92×10^{-5}
rs76970177	*SEMA3C*; *LOC105369146*	7	81364751	1.48 (1.24–1.77)	1.42×10^{-5}	1.49 (1.25–1.78)	9.78×10^{-6}
rs79617578	*SEMA3C*; *LOC105369146*	7	81376899	1.44 (1.21–1.72)	4.93×10^{-5}	1.45 (1.21–1.73)	4.20×10^{-5}
rs72508697	*SEMA3C*; *LOC105369146*	7	81377136	1.44 (1.21–1.72)	4.93×10^{-5}	1.45 (1.21–1.73)	4.20×10^{-5}
rs73492791	*NANS*	9	98071643	0.65 (0.53–0.80)	2.72×10^{-5}	0.65 (0.53–0.80)	3.13×10^{-5}
rs3780471	*TRIM14*	9	98085269	0.65 (0.54–0.79)	2.09×10^{-5}	0.65 (0.53–0.79)	1.59×10^{-5}
rs7137657	*LINC02408*; *DYRK2*	12	67605696	1.68 (1.32–2.12)	1.79×10^{-5}	1.69 (1.33–2.15)	1.97×10^{-5}
rs1579655	*LOC101928617*; *CLLU1OS*	12	92242372	0.72 (0.61–0.84)	3.25×10^{-5}	0.71 (0.61–0.84)	2.51×10^{-5}
rs1866248	*LOC101928617*; *CLLU1OS*	12	92242857	0.72 (0.61–0.84)	3.25×10^{-5}	0.71 (0.61–0.84)	2.51×10^{-5}
rs7303321	*LOC101928617*; *CLLU1OS*	12	92246410	0.72 (0.62–0.84)	3.61×10^{-5}	0.72 (0.61–0.84)	3.06×10^{-5}
rs10859287	*LOC101928617*; *CLLU1OS*	12	92248968	0.7 (0.59–0.82)	7.52×10^{-6}	0.69 (0.59–0.81)	5.72×10^{-6}
rs10745588	*LOC101928617*; *CLLU1OS*	12	92250325	0.69 (0.59–0.81)	6.83×10^{-6}	0.69 (0.59–0.81)	5.26×10^{-6}
rs872969	*LOC101928617*; *CLLU1OS*	12	92252224	0.71 (0.60–0.83)	1.60×10^{-5}	0.7 (0.60–0.82)	1.23×10^{-5}
rs10777386	*LOC101928617*; *CLLU1OS*	12	92253150	0.71 (0.60–0.83)	1.60×10^{-5}	0.7 (0.60–0.82)	1.23×10^{-5}
rs2805890	*LOC101928617*; *CLLU1OS*	12	92254617	0.71 (0.60–0.83)	1.60×10^{-5}	0.7 (0.60–0.82)	1.23×10^{-5}
rs1545152	*LOC101928617*; *CLLU1OS*	12	92261661	0.71 (0.61–0.83)	2.37×10^{-5}	0.71 (0.60–0.83)	1.65×10^{-5}
rs6538323	*LOC101928617*; *CLLU1OS*	12	92264008	0.71 (0.61–0.83)	2.37×10^{-5}	0.71 (0.60–0.83)	1.65×10^{-5}
rs6538324	*LOC101928617*; *CLLU1OS*	12	92264747	0.71 (0.61–0.83)	2.37×10^{-5}	0.71 (0.60–0.83)	1.65×10^{-5}
rs11106481	*LOC101928617*; *CLLU1OS*	12	92265670	0.71 (0.61–0.83)	2.37×10^{-5}	0.71 (0.60–0.83)	1.65×10^{-5}
rs78518801	*CCDC169-SOHLH2*; *SOHLH2*	13	36188063	1.86 (1.43–2.41)	2.94×10^{-6}	1.8 (1.38–2.34)	1.34×10^{-5}
rs79882008	*CCDC169-SOHLH2*; *SOHLH2*	13	36197158	1.82 (1.40–2.35)	5.24×10^{-6}	1.72 (1.32–2.22)	4.84×10^{-5}
rs2322895	*CCDC169-SOHLH2*; *SOHLH2*	13	36198931	1.82 (1.41–2.35)	3.88×10^{-6}	1.73 (1.33–2.24)	3.52×10^{-5}
rs78972162	*CCDC169-SOHLH2*; *SOHLH2*	13	36199197	1.82 (1.41–2.35)	3.88×10^{-6}	1.73 (1.33–2.24)	3.52×10^{-5}
rs2149425	*CCDC169-SOHLH2*; *SOHLH2*	13	36201154	1.79 (1.39–2.31)	6.89×10^{-6}	1.71 (1.32–2.21)	4.26×10^{-5}
rs17053630	*CCDC169-SOHLH2*; *SOHLH2*	13	36202364	1.79 (1.39–2.31)	6.89×10^{-6}	1.71 (1.32–2.21)	4.26×10^{-5}
rs77706449	*CCDC169-SOHLH2*; *SOHLH2*	13	36206125	1.78 (1.38–2.30)	7.95×10^{-6}	1.7 (1.32–2.20)	4.85×10^{-5}
rs74694612	*CCDC169-SOHLH2*; *SOHLH2*	13	36209455	1.82 (1.41–2.34)	4.48×10^{-6}	1.72 (1.33–2.23)	4.00×10^{-5}
rs117293024	*KCNH5*	14	62952042	1.77 (1.36–2.32)	2.87×10^{-5}	1.78 (1.35–2.35)	4.66×10^{-5}

(Continued)

Table S3: *Continued*

SNPs	Gene	Chr	BP	OS		PFS	
				HR (95% CI)	*p*	HR (95% CI)	*p*
rs76608343	*GINS3*	16	58395747	1.37 (1.18–1.58)	2.70×10^{-5}	1.36 (1.18–1.57)	3.28×10^{-5}
rs4888508	*CNTNAP4*	16	76438786	1.36 (1.18–1.56)	1.58×10^{-5}	1.33 (1.16–1.53)	4.79×10^{-5}
rs12940675	*RAI1*	17	17747900	1.76 (1.36–2.28)	1.96×10^{-5}	1.76 (1.35–2.30)	3.06×10^{-5}
rs12945601	*RAI1*	17	17750097	1.76 (1.36–2.28)	1.96×10^{-5}	1.76 (1.35–2.30)	3.06×10^{-5}
rs941446	*SMCR5*	17	17776959	1.86 (1.42–2.42)	5.94×10^{-6}	1.8 (1.37–2.36)	2.20×10^{-5}
rs61377283	*MIR4318*; *MIR924HG*	18	38870239	0.76 (0.66–0.87)	$4.63 \times 10^{-5} \times 10^{-5}$	0.75 (0.65–0.85)	2.04×10^{-5}
rs11876568	*MIR4318*; *MIR924HG*	18	38870468	0.76 (0.66–0.87)	4.63×10^{-5}	0.75 (0.65–0.85)	2.04×10^{-5}
rs4133291	*MIR4318*; *MIR924HG*	18	38898280	0.75 (0.66–0.86)	2.62×10^{-5}	0.73 (0.64–0.84)	8.18×10^{-6}
rs56407818	*LOC100420587*; *LINC00906*	19	28788472	0.65 (0.53–0.79)	2.30×10^{-5}	0.65 (0.53–0.80)	3.12×10^{-5}
rs55849754	*LOC100420587*; *LINC00906*	19	28788758	0.65 (0.53–0.79)	2.30×10^{-5}	0.65 (0.53–0.80)	3.12×10^{-5}
rs17498203	*LOC100420587*; *LINC00906*	19	28788827	0.65 (0.53–0.79)	2.30×10^{-5}	0.65 (0.53–0.80)	3.12×10^{-5}
rs7247294	*LOC100420587*; *LINC00906*	19	28789022	0.65 (0.54–0.79)	2.08×10^{-5}	0.66 (0.54–0.80)	2.75×10^{-5}
rs12151001	*LOC100420587*; *LINC00906*	19	28792479	0.65 (0.53–0.79)	2.30×10^{-5}	0.65 (0.53–0.80)	3.12×10^{-5}
rs73029061	*LOC100420587*; *LINC00906*	19	28793073	0.65 (0.53–0.79)	2.30×10^{-5}	0.65 (0.53–0.80)	3.12×10^{-5}
rs6035474	*PDYN–AS1*; *STK35*	20	2061818	1.67 (1.32–2.11)	1.54×10^{-5}	1.63 (1.29–2.06)	4.51×10^{-5}
rs6136889	*PDYN–AS1*; *STK35*	20	2064746	1.69 (1.35–2.13)	6.64×10^{-6}	1.66 (1.32–2.09)	1.71×10^{-5}
rs147068033	*D21S2088E*; *LINC01689*	21	23637454	1.37 (1.19–1.59)	1.94×10^{-5}	1.37 (1.19–1.59)	1.87×10^{-5}

SNP: single nucleotide polymorphism, Chr: chromosome, BP: biological position, OS: overall survival, PFS: progression–free survival, HR: hazard ratio, CI: confidence interval.
